# Assembly of the Type II Secretion System such as Found in *Vibrio cholerae* Depends on the Novel Pilotin AspS

**DOI:** 10.1371/journal.ppat.1003117

**Published:** 2013-01-10

**Authors:** Rhys A. Dunstan, Eva Heinz, Lakshmi C. Wijeyewickrema, Robert N. Pike, Anthony W. Purcell, Timothy J. Evans, Judyta Praszkier, Roy M. Robins-Browne, Richard A. Strugnell, Konstantin V. Korotkov, Trevor Lithgow

**Affiliations:** 1 Department of Biochemistry and Molecular Biology, Monash University, Melbourne, Australia; 2 Victorian Bioinformatics Consortium, Monash University, Melbourne, Australia; 3 Department of Molecular and Cellular Biochemistry and Center for Structural Biology, University of Kentucky, Lexington, Kentucky, United States of America; 4 Department of Microbiology and Immunology, The University of Melbourne, Melbourne, Australia; 5 Monash Institute of Medical Research, Melbourne, Australia; 6 Murdoch Childrens Research Institute, Royal Children's Hospital, Melbourne, Australia; Osaka University, Japan

## Abstract

The Type II Secretion System (T2SS) is a molecular machine that drives the secretion of fully-folded protein substrates across the bacterial outer membrane. A key element in the machinery is the secretin: an integral, multimeric outer membrane protein that forms the secretion pore. We show that three distinct forms of T2SSs can be distinguished based on the sequence characteristics of their secretin pores. Detailed comparative analysis of two of these, the *Klebsiella*-type and *Vibrio*-type, showed them to be further distinguished by the pilotin that mediates their transport and assembly into the outer membrane. We have determined the crystal structure of the novel pilotin AspS from *Vibrio cholerae*, demonstrating convergent evolution wherein AspS is functionally equivalent and yet structurally unrelated to the pilotins found in *Klebsiella* and other bacteria. AspS binds to a specific targeting sequence in the *Vibrio*-type secretins, enhances the kinetics of secretin assembly, and homologs of AspS are found in all species of *Vibrio* as well those few strains of *Escherichia* and *Shigella* that have acquired a *Vibrio*-type T2SS.

## Author Summary

The type 2 secretion system (T2SS) is a sophisticated, multi-component molecular machine that drives the secretion of fully-folded protein substrates across the bacterial outer membrane. In *Vibrio cholerae*, for example, the T2SS mediates the secretion of cholera toxin. We find that there are three distinct forms of T2SS, based on the sequence characteristics of the secretin. A targeting paradigm, developed for the *Klebsiella*-type secretin PulD, could not previously be applied to the T2SS in *Vibrio cholerae* and many other bacterial species whose genomes encode no homolog of the crucial targeting factor PulS (also called OutS, EtpO or GspS). Using bioinformatics we find, remarkably, that these bacteria have instead evolved a structurally distinct protein to serve in place of PulS. We crystallized and solved the structure of this distinct factor, AspS, measured its activity in novel assays for T2SS assembly, and show that the protein is essential for the function of the *Vibrio*-type T2SS. A structural homolog of AspS found here in *Pseudomonas* suggests widespread use of the pilotin-secretin targeting paradigm for T2SS assembly.

## Introduction

Bacterial outer membranes incorporate proteins of at least three well-characterized architectures: β-barrel proteins, lipoproteins and secretins. The integral membrane proteins having a β-barrel architecture are targeted to the outer membrane and assembled by the β-barrel assembly machinery, the BAM complex [1]–[3]. Lipoproteins, anchored in the outer membrane by covalently attached lipid modifications, are inserted into the outer membrane by the receptor LolB after being ferried across the periplasm by factors of the Lol machinery [4], [5]. Secretins are integral proteins which assemble to form multimeric secretion channels in the outer membrane, with examples including outer membrane proteins of the Type II Secretion Systems (T2SS) and Type III Secretion Systems (T3SS), Type IV fimbrae and the filamentous phage extrusion machinery [6]–[9]. In the case of the T2SS, the secretin multimer in the outer membrane docks onto a platform of inner membrane proteins that energize its function in the selection and/or secretion of one or a few substrate proteins across the outer membrane into the external *milieu* [10]–[12].

The archetypal T2SS secretin is the outer membrane protein PulD from *Klebsiella oxytoca* [13], [14]. The PulD polypeptide has three identifiable domains: the N-domain that docks it to the inner membrane components of the T2SS, the secretin domain (also called the C-domain) responsible for multimerization, and the S-domain which is critical for PulD to engage the targeting pathway that will deliver it to the outer membrane [14]–[18]. This targeting of PulD depends on the action of a lipoprotein chaperone, which carries in its structure the determinants to be recognized by the Lol machinery receptor LolB [5], [19]. The chaperone targeting PulD to the outer membrane is referred to as a pilotin and, in *K. oxytoca*, the pilotin is called PulS. PulS is the progenitor member of the PulS-OutS family of proteins found in diverse species of γ-proteobacteria. For example, in *Dickeya dadantii* and *Pectobacterium chrysanthemi* the homologous protein is called OutS [20] and in enterohemorrhagic *Escherichia coli* O157 strains the homologous protein is called EtpO [21]. All of these proteins are conserved in sequence features established for the PulS-OutS family of proteins (pfam09691), and for three examples: from *Klebsiella oxytoca* [18], *Dickeya dadantii* [22] and *E. coli* O157:H7 (PDB 3SOL), the proteins have been crystallized and the structures are highly conserved.

Pilotins of the PulS-OutS family function by directly binding to a short segment within the S-domain of their appropriate secretin [17], [18], [22]. Mapping experiments using affinity chromatography and structural analysis show that the S-domain of PulD is natively-disordered but, that binding to PulS induces folding, complementarity and fit in the S-domain:PulS complex [17], [18]. In crystal structures of the homologous OutS pilotin, an 18 residue segment from the S-domain folds into a well-ordered α-helix once captured by the pilotin [22]. The S-domain is both necessary and sufficient for secretin targeting: mutagenesis of this region of PulD renders it incapable of reaching the outer membrane, while experiments in which the segment from PulD was transferred into the S-domain of the pIV secretin of the filamentous phage extrusion machinery rendered pIV secretin dependent on PulS for targeting to the outer membrane [15], [17], [20].

Recent characterization of the T2SS in enteropathogenic *E. coli* O127:H6 str. E2348/69 (EPEC) revealed its function in secreting the protein substrate SslE [23]. SslE is found in very few strains of *E. coli*, but a homologous protein AcfD is widely distributed in species of *Vibrio* [24] leading to the hypothesis that organisms like *Vibrio cholerae* use the T2SS to secrete both cholera toxin and SslE/AcfD [23], with the expression of the genes encoding cholera toxin and AcfD known to be co-regulated [25]. In EPEC, SslE secretion is required for biofilm formation [23] and, similarly, in *V. cholerae* AcfD secretion is required for intestinal colonization [25]. It is unclear how the T2SS secretin is assembled in the organisms that secrete SslE/AcfD: EPEC does not encode the pilotin EtpO, and *V. cholerae* genomes have not been reported to encode any members of the PulS-OutS family of proteins.

We sought to better understand how the T2SS secretin is assembled into a functional multimer by EPEC. Hidden Markov model analysis of the genome identified a protein called YacC which,while having only 21% sequence identity to the previously characterized *E. coli* protein EtpO, has the conserved features of the PulS-OutS family of proteins. However, YacC does not function as a pilotin to transport the GspD secretin to the outer membrane in EPEC, as judged by kinetic analysis of protein trafficking and functional assays of T2SS-dependent secretion of SslE. Instead, we found that a distinct lipoprotein AspS (*A*lternate *s*ecretin *p*athway subunit *S*) functions as the pilotin for GspD in EPEC. In an example of convergent evolution to a common function, the crystal structure of AspS shows it to have no structural similarity whatsoever to the PulS-OutS family of proteins. Biochemical analysis demonstrates that AspS binds to an S-domain sequence in the *Vibrio*-type secretins, with sequence analysis distinguishing the S-domains of the *Klebsiella*-type and *Vibrio*-type secretins. Taken together these findings reveal that distinct classes of T2SS secretins can be recognized: one represented by the *Klebsiella* PulD which make use of PulS-OutS pilotins, and one represented by the *Vibrio* EpsD/GspD that makes use of AspS pilotins. We suggest that *E. coli* pathotypes that have acquired the *Klebsiella*-type secretin depend on PulS-OutS pilotins such as EtpO, whereas *E. coli* pathotypes that have acquired the *Vibrio*-type secretin depend on the AspS pilotin to assemble a functional T2SS.

## Results

### PulS-OutS family HMM analysis detects YacC in the genome of EPEC 2348/69

**Figure 1:**
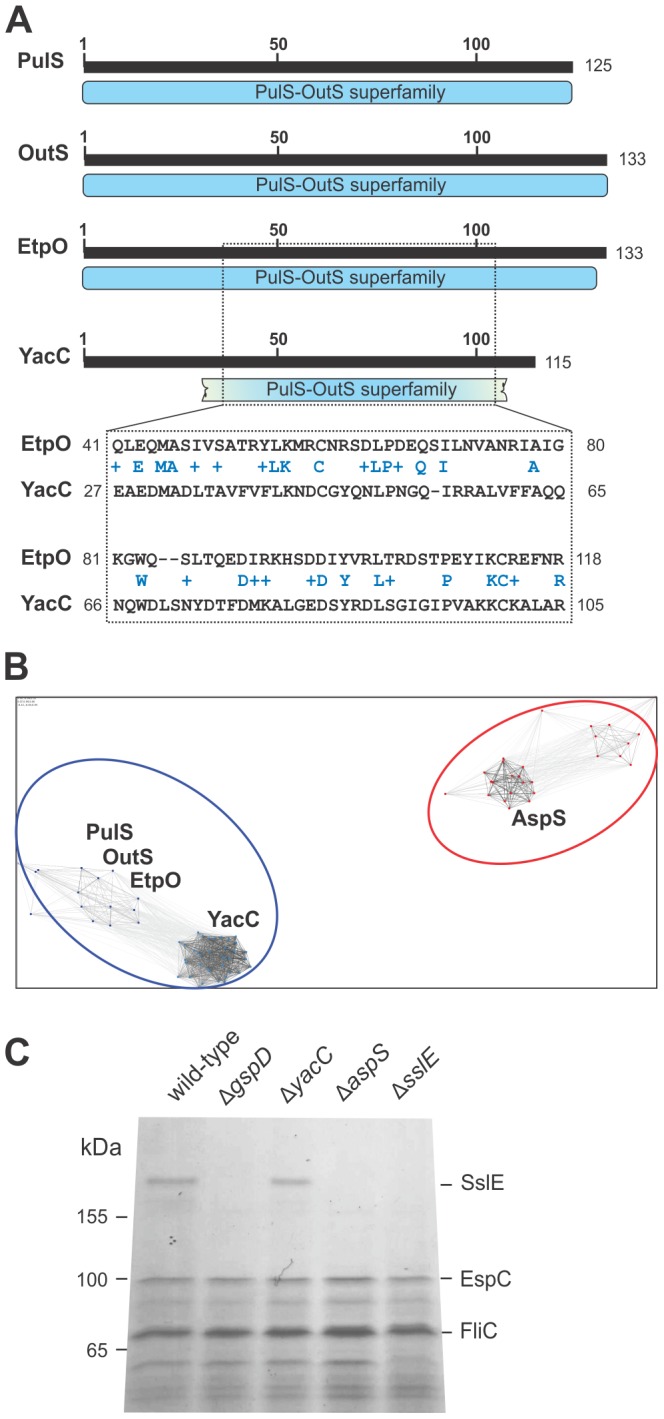
YacC is a novel member of the PulS-OutS family of proteins. (A) The conserved domain architecture tool (CDART) was used to map the regions of PulS from *Klebsiella oxytoca* 10-5250 (EHT07154.1), OutS from *Dickeya dadantii* 3937 (YP_003883937.1), EtpO from Shiga toxin-producing *E. coli* (STEC) O157:H7 (CAA70966.1) and YacC (CAS07673.1) from EPEC. Numbers refer to the amino acids of each protein sequence, and the broken blue bar denotes that the N-terminal and C-terminal residues of YacC diverge from the consensus features of the PulS-OutS family. Pairwise sequence alignment over 80 residues showing similarity of the previously characterized EtpO (CAA70966.1) and YacC (CAS07673.1). Identical residues are highlighted between the two sequences, and conserved substitutions are shown (+). (B) CLANS analysis graphically depicts homology in large datasets of proteins, utilizing all-against-all pairwise BLAST to cluster representations (colored dots) of individual protein sequences in three-dimensional space. Lines are shown between the most similar sequences, with an E-value cut-off of 1e−5. The analysis shows that proteins from diverse species cluster into two groups: the PulS/OutS group and YacC-related proteins (blue), and the AspS-related proteins (red). There are numerous relationships between the PulS/OutS proteins and YacC proteins, but no relationship links these to the AspS group of proteins. (C) Wild-type EPEC (WT), and the indicated mutants of EPEC were grown in culture and post-cell supernatants containing secreted proteins (200 µg of protein) were analyzed by SDS-PAGE and Coomassie blue staining. Mass spectrometry was used to identify SslE, FliC and EspC, consistent with a previous study [72].

The Pfam definition of the PulS-OutS protein family was initially derived from conserved domain architecture statistics [26] of four protein sequences: PulS from *Klebsiella*, OutS from *Dickeya*, OutS from *Pectobacterium*, and EtpO from *E. coli* O157:H7. The current version of Pfam lists 174 non-redundant PulS-OutS protein family members that were identified from genomic sequence data, and these are defined as containing the conserved domain architecture of the PulS-OutS protein family, consistent with that of PulS, OutS and EtpO (Figure 1A).

In order to have a highly-sensitive tool to detect distant forms of the PulS-OutS protein family encoded in the EPEC genome, we constructed a hidden Markov model and searched the sequence data with a threshold cut-off E value of 10e−3. We observed a single, statistically-significant hit (E value = 1.10e−41) to the protein YacC that has only limited (21%) sequence identity to the *E. coli* pilotin EtpO, and conforms partly to the conserved domain architecture of the PulS-OutS protein family (Figure 1A). In addition, the hidden Markov model analysis assigned a low confidence score (E value = 4.20e−03) to a previously uncharacterized protein, YghG. The sequence match is not statistically significant, but YghG is coincidentally encoded from a gene within the transcriptional unit coding for the T2SS of EPEC [23], [27], and shows the sequence characteristics of a lipoprotein: the sequence analysis tool LipoP [28] predicts a signal peptidase II cleavage sequence which would yield an N-terminus commencing with the sequence CASHN in a matured lipoprotein. For reasons described later, we refer to YghG and its apparent homologs in species of *Vibrio* and *Shigella* as AspS (*A*lternate general *s*ecretion *p*rotein subunit S).

The T2SS from EPEC and *V. cholerae* secrete similar substrates [23], and yet no PulS-OutS pilotin has been detected previously in genome sequences of *V. cholerae*, and no high-scoring sequences were detected with our HMM search of the genome of the type strain *V. cholerae* O1 biovar El Tor N16961. However, using a BLAST search with AspS as a query, the protein sequence VC1703 (NP_231339.1) was detected in this *V. cholerae* genome and found to have very high (52%) sequence similarity to AspS from EPEC (Figure S1A). AspS-related protein sequences were found in all strains of *V. cholerae* and other species of the genus *Vibrio*, and in *Shigella boydii* ATCC 9905 and *Shigella* sp. D9. All of these bacteria have clearly recognizable operons that would encode a T2SS, and in EPEC, *Shigella boydii* ATCC 9905 and *Shigella* sp. D9 the gene encoding AspS is embedded within that operon (Figure S1B).

To demonstrate and characterize the relationship of the PulS-OutS sequences to each other and to the groups of YacC and AspS proteins detected in BLAST searches, we made use of CLuster ANalysis of Sequences (CLANS) [29]. The analysis defined YacC and related proteins from other species as being a distinct grouping, and showed that this group of proteins is related to the PulS-OutS family of pilotins. It also showed that there was no statistically supported relationship between the AspS group of proteins and the PulS-OutS family of proteins (Figure 1B). Thus, EPEC encodes two previously uncharacterized proteins: one (YacC) with the sequence characteristics of previously characterized T2SS pilotins and another, which is an unrelated lipoprotein (AspS).

To determine whether loss of either YacC or AspS has phenotypic consequences to T2SS function, we monitored the secretion of SslE, the major substrate of the T2SS in EPEC [23]. SslE is a ∼165 kDa protein with signature sequences for the M60-like class of enhancin metalloproteases, and the same domain features are conserved in the AcfD proteins found in species of *Vibrio* and *Shigella* [24]. The parental EPEC strain and mutants lacking either GspD, YacC or AspS were grown and the “secretome” of the culture supernatants evaluated by SDS-PAGE and Coomassie blue staining. In all strains, the dominant secreted proteins EspC and FliC were unaltered. The identity of SslE was confirmed by its absence from Δ*sslE* mutants and by mass spectrometry of the protein present in the secretome of wild-type EPEC. While SslE is present in the secretome of Δ*yacC* mutants, it is not secreted by the mutants lacking AspS (Figure 1C).

### Assays to monitor the kinetics of assembly of the T2SS secretin into outer membranes

**Figure 2:**
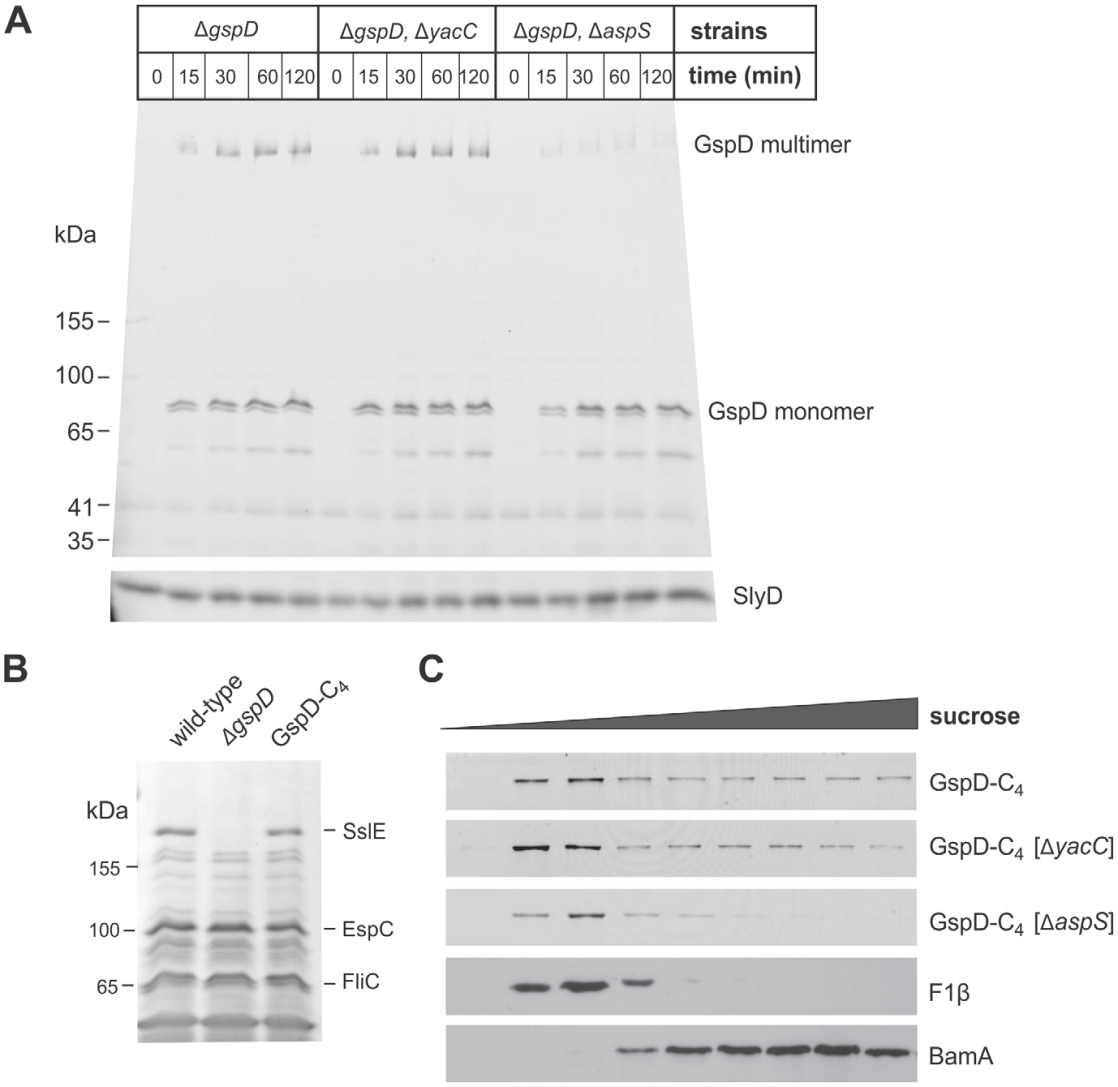
The kinetics of assembly, measured *in vivo*, for GspD in EPEC. (A) The indicated strains of EPEC, complemented with the plasmid encoding GspD-C_4_ were cultured in medium to an OD_600_∼0.6 and arabinose was then added to the culture (0.1%, final concentration). At the indicated time-point cell extracts were prepared from the culture, resuspended in sample buffer containing Lumio reagent and analysed by SDS-PAGE. The polyacrylamide gels were then imaged by fluorimetry (Argon Blue 488 nm laser and 520 nm BP40 filter). Positions of molecular weight markers and the 21 kDa protein SlyD are indicated. (B) Wild-type EPEC (WT), Δ*gspD* mutant EPEC, and the Δ*gspD* mutant EPEC complemented with the plasmid encoding GspD-C_4_ were grown in culture and post-cell supernatants containing secreted proteins (200 µg of protein) were analyzed by SDS-PAGE and Coomassie blue staining. (C) Strains of EPEC: Δ*gspD*, Δ*gspD*Δ*yacC* or Δ*gspD*Δ*aspS*, were complemented with the plasmid encoding GspD-C_4_ under control of the *tet* promoter and were cultured to an OD_600_∼1.0, extracted and then fractionated by sucrose density centrifugation. Identical samples were analysed by SDS-PAGE for detection of GspD multimers with Lumio reagent and immunoblotting for the outer membrane protein BamA and the inner membrane β-subunit of the F_1_F_o_-ATP synthase (F_1_β).

To determine if either YacC or AspS functioned as a pilotin for the assembly of GspD secretin, we engineered three deletion mutants in EPEC: aΔ*gspD* mutant, a Δ*gspD*,Δ*yacC* double mutant, and a Δ*gspD*,Δ*aspS* double mutant, and complemented each with a plasmid (strains and plasmids are documented in Table S1) carrying the *gspD* gene from EPEC under the control of an arabinose-inducible promoter, and modified it so that the GspD protein has a tetra-cysteine (“FlAsH”) tag at its C-terminus. This modified GspD is hereafter referred to as GspD-C_4_. This provided a means to selectively label GspD monomers and multimers after a rapid SDS-PAGE-based assay of total cell extracts using FlAsH Tag technology [30]. In these complemented cells, GspD-C_4_ expression was observed labelled with Lumio reagent within 15 minutes of induction with arabinose. The monomeric form of GspD-C_4_ is detected at early time-points, and multimers of GspD-C_4_ form with a slight delay in kinetics (Figure 2A). In what proved to be a convenient internal loading control, the endogenous metallo-chaperone SlyD reacts with the Lumio reagent. EPEC mutants lacking YacC assembled GspD-C_4_ with the same kinetics as the complemented “wild-type” strain. By contrast, while GspD-C_4_ monomers were expressed in Δ*aspS* mutants, in the absence of AspS the multimerization of GspD-C_4_ was greatly retarded (Figure 2A).

The FlAsH-tagged GspD-C_4_ was assembled into a functional form, since Δ*gspD* mutants expressing GspD-C_4_ secrete the T2SS substrate SslE at wild-type levels (Figure 2B). We therefore sought to demonstrate that the multimers of GspD were selectively present in the outer membrane by using sucrose density fractionation. It was consistently clear that GspD-C_4_ multimers were in the outer membrane fractions of wild-type and Δ*yacC* mutants, and were not present in the outer membranes of EPEC lacking AspS (Figure 2C). However, the varying amounts of multimers seen in the inner membrane fractions of the processed fractions were evident even in the Δ*aspS* mutant fractions (Figure 2C), when they were not evident in the rapidly prepared extracts from intact cells (Figure 2A). Thus, use of this EPEC system for sub-cellular fractionation was hampered by ongoing over-expression of GspD-C_4_ during sample processing, leading to ill-defined amounts of GspD-C_4_ multimers in the inner membrane fractions.

**Figure 3:**
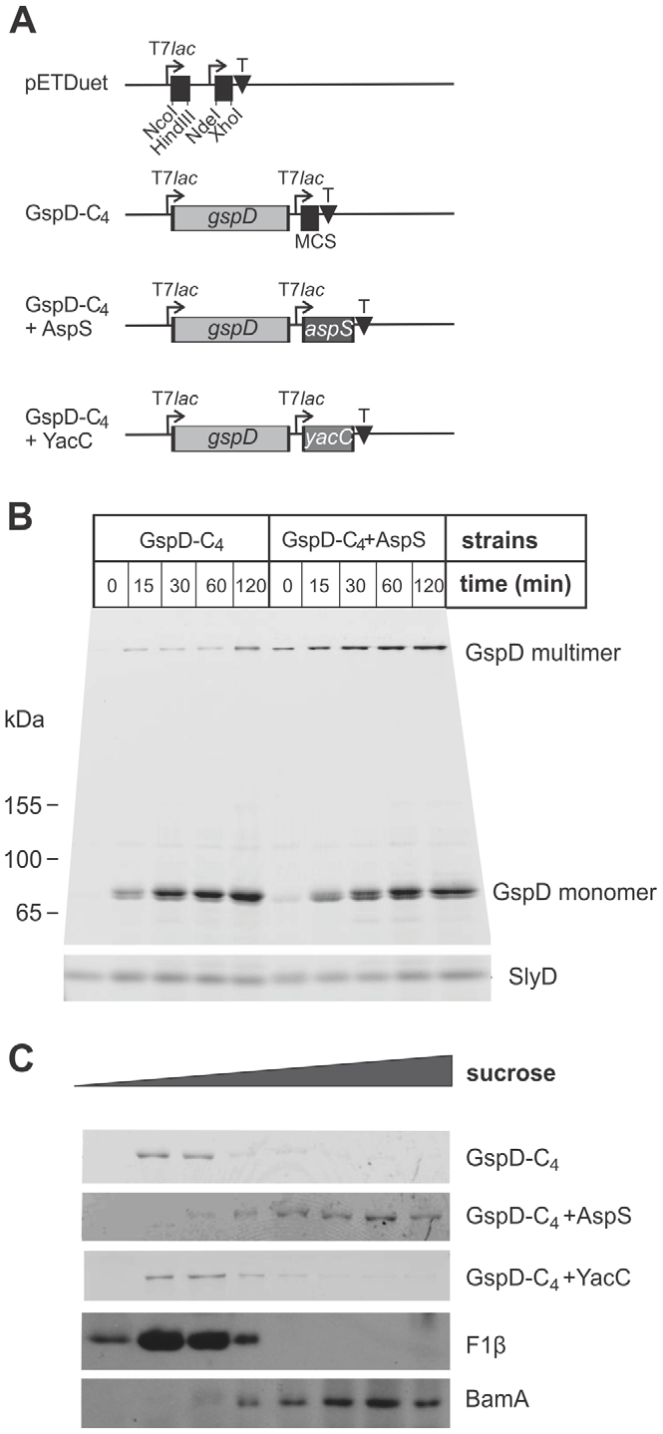
Subcellular targeting of GspD to the outer membrane depends on AspS. (A) The expression cassette in pETDuet plasmids GspD-C_4_, GspD-C_4_+AspS and GspD-C_4_+YacC are represented diagrammatically. The pETDuet-1 vector (Novagen) has two multi-cloning sites (MCS) represented as black squares, and cloning into the NcoI and NdeI sites is optimal with respect to the ribosome-binding sites: NcoI and HindIII sites were used to clone the open-reading frame corresponding to GspD-C_4_; NdeI and XhoI sites were used to clone the open-reading frame corresponding to AspS and YacC. The T7 terminator sequence (T) in the plasmid is represented by a black triangle. (B) *E. coli* BL21(DE3)(Δ*gspD*,Δ*aspS*) complemented with pETDuet (GspD-C_4_) or pETDuet (GspD-C_4_+AspS) were cultured to an OD_600_ of ∼0.6 and IPTG was added to the culture (0.1 mM, final concentration). At the indicated time-point cell extracts were prepared from the cultures using Lumio analysed by SDS-PAGE and imaged by fluorimetry. (C) The strains described above were cultured to an OD_600_∼1, extracted and then fractionated by sucrose density centrifugation. Replicate samples were analysed by SDS-PAGE for detection of GspD multimers with Lumio reagent and immunoblotting for BamA and F_1_β.

We established a second assembly assay system using the model *E. coli* strain BL21(DE3). The genes *gspD* and *aspS* were deleted in this strain background, the coding sequences for GspD-C_4_, with or without putative pilotins, were cloned into a pETDuet vector (Figure 3A), and the plasmids transformed into *E. coli* BL21(DE3)(Δ*gspD*,Δ*aspS*). Using minimal induction of expression (see Methods) even after 120 minutes of induction we consistently observed only a very low amount of GspD multimer in the absence of AspS (Figure 3B), but a much more rapid conversion of the monomer to GspD multimer in the presence of AspS. Importantly, sucrose density gradients revealed that in the absence of AspS all detectable GspD was associated with the inner membranes, co-migrating with the marker protein F_1_β (Figure 3C). This is consistent with previous observations that the *K. oxytoca* secretin PulD assembles and inserts into the inner membrane in the absence of its pilotin PulS [31]. In the presence of AspS, all of the GspD multimer was detected in the outer membrane fractions (Figure 3C). We conclude that AspS is the pilotin for the EPEC secretin GspD. By contrast, co-expression of YacC had no effect on trafficking and assembly of GspD. Bioinformatics analyses do not detect a second T2SS secretin encoded in the EPEC genome (data not shown); thus, either YacC functions as a pilotin for an unrelated group of secretins, or it performs a fundamentally different function.

### Distinct types of T2SS secretins are defined by the sequence characteristics of the S-domain

**Figure 4:**
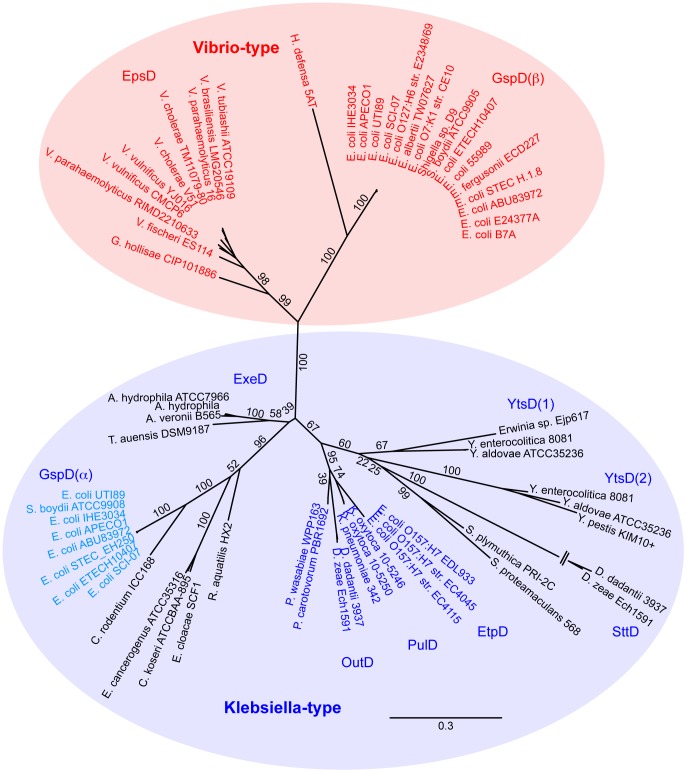
Phylogenetic analysis reveals distinct types of T2SS secretins. Phylogenetic tree reconstruction was performed with PhyML v3.0 [60] using 500 bootstrap calculations and shown as percentage values (for further details see Methods). Based on the strong statistical support in the division, the *Vibrio*-type and *Klebsiella* type secretin sub-families are highlighted with colour. These have also been labelled as “GspDα” and “GspDβ” in accordance with a new nomenclature recently proposed for ETEC str. H10407 [35]. The branch to the secretin SttD in *Dickeya* spp. is not shown to full scale. For a full list of the sequence accession numbers see Table S2.

To our knowledge there has not previously been a systematic assessment of the T2SS secretin family, yet the finding of distinct types of pilotins raised the prospect that distinct types of secretins exist. A phylogenetic analysis of all of the known T2SS secretins demonstrated three groups, with one of these consisting of sequences from species of *Pseudomonas*, *Xanthomonas* and *Legionella* forming a well-supported single long branch only distantly related to the remaining more similar sequences (data not shown). From within this set of “*Pseudomonas*-type” secretins, there are documented accounts of unusual modes of assembly and action [32]–[34] and this divergent group was removed from further analysis, in order to best assess the relationships of the secretins found in various pathotypes of *E. coli* and the well-studied secretins from *Klebsiella* and related organisms. The in-depth analysis revealed that two well-supported sub-families are clearly identified: (i) a group that included both the EpsD proteins from *Vibrio* and the GspD protein from EPEC, *Shigella* and a few pathotypes of *E. coli*, and (ii) the “*Klebsiella*-type” secretins that included the characterized proteins PulD and OutD from species of *Klebsiella*, *Dickeya* and *Pectobacterium* together with the related group of EtpD secretins (Figure 4). The secretins found in pathotypes of *E. coli* are not all homologous: EtpD and a group of secretins referred to in the literature as “GspD” are two sets of secretins, found within the overall *Klebsiella*-type. A third, and distinct, set is represented by the secretin found in EPEC (unfortunately also referred to as “GspD”) which groups together with the “*Vibrio*-type secretins” such as EpsD from *V. cholerae*. A good example of this can be seen in the secretins, GspD(α) and GspD(β), recently described in enterotoxigenic *E. coli* (ETEC) str. H10407 [35] and shown in Figure 4. A revision of the *E. coli* T2SS nomenclature is indicated given that the *E. coli* GspD(α) secretin is more closely related to the *E. coli* EtpD secretins than it is to the *E. coli* GspD(β) secretin (Figure 4).

**Figure 5:**
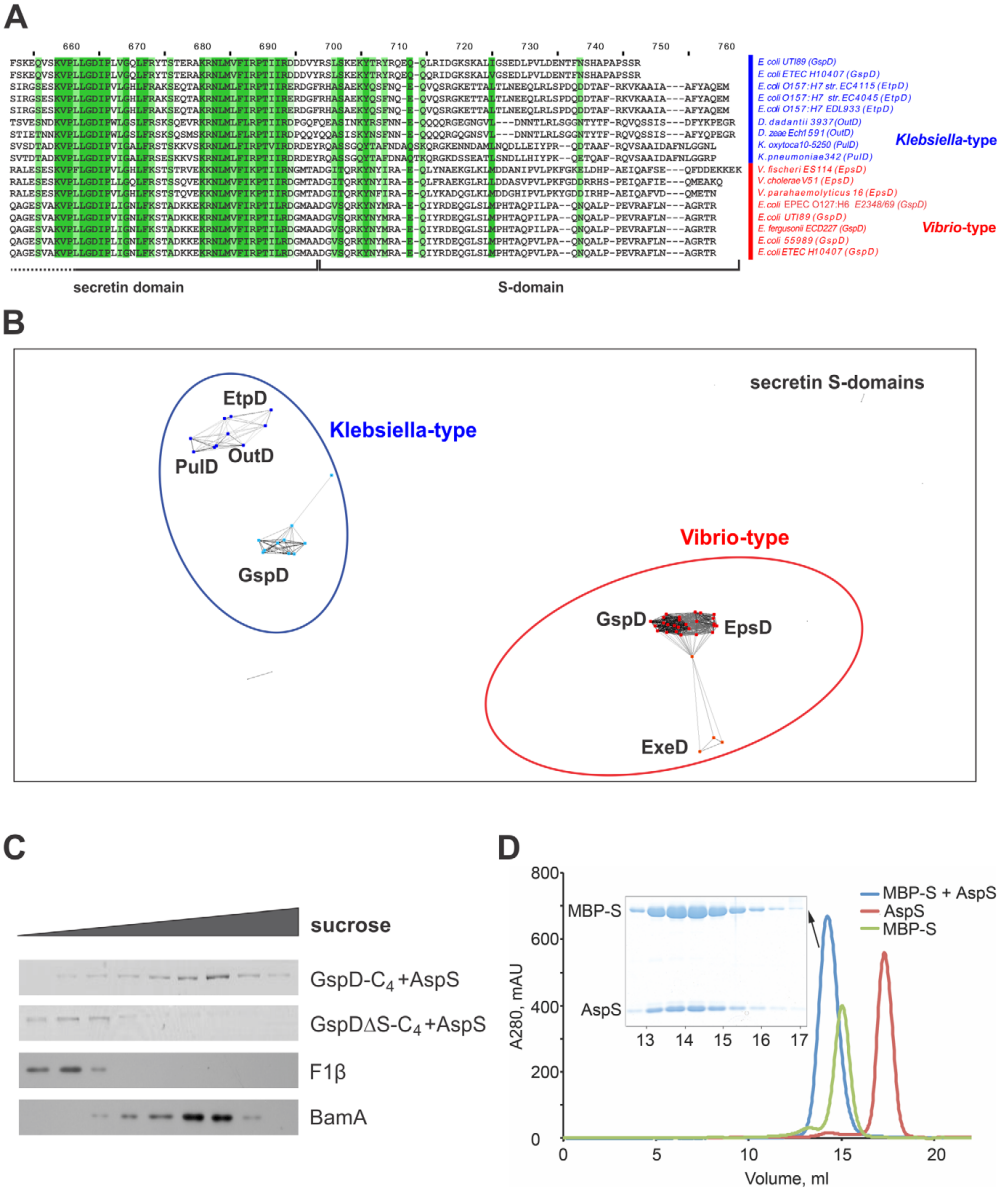
The S-domains of *Vibrio*-type secretins have diagnostic sequence features. (A) Alignment of a representative subset of the secretin sequences used in this study to demonstrate sequence conservation (darker to lighter shades of green represent higher to lower levels of sequence conservation). Accession numbers for all secretins investigated in this study are given in Table S2. (B) S-domain sequences from the secretins were subject to CLANS analysis [29]. The position corresponding to each S-domain sequence from the *Vibrio*-type EpsD and GspD proteins is represented by red dots, ExeD by orange dots and the group circled in red. The position corresponding to each sequence from the *Klebsiella*-type PulD, OutD, EtpD and GspD proteins is colour-coded in blue. The connections shown represent an E-value cut-off of 1e−10. (C) *E. coli* BL21(DE3)(Δ*gspD*,Δ*aspS*) complemented with either pETDuet (GspD-C_4_+AspS) or pETDuet (GspDΔS-C_4_+AspS) were cultured to an OD_600_ of ∼0.6 and IPTG was added to the culture (0.1 mM, final concentration). At the indicated time-point cell extracts were prepared from the cultures and incubated with a modified sample buffer containing Lumio reagent, analysed by SDS-PAGE and imaged by fluorimetry. (D) Size-exclusion chromatography profiles of the purified AspS (red), the purified MBP-S-domain fusion (green) and the complex of AspS and MBP-S-domain fusion (blue) on a Superdex200 column. An SDS-PAGE gel of the peak fractions of AspS-MBP-S-domain complex shows an approximately stoichiometric ratio of the two proteins. A280, absorbance at 280 nm; mAU, milli absorbance units. Figure S5 shows the results of the control experiment, where AspS and MBP without S-domain do not interact.

In studies on *K. oxytoca* PulD, the S-domain has been defined as the C-terminal 60 amino acids, a region immediately after the defining secretin domain (Figure 5A). Biochemical analysis [17], [18] has demonstrated that the region corresponding to the S-domain is necessary and sufficient for pilotin binding. In order to evaluate how well conserved the S-domain sequences of the *Vibrio*-type secretins might be, the sequence collection was subject to CLANS. The analysis demonstrated that statistically significant (E-value = 1e−10) relationships exist in the *Vibrio*-type secretin S-domain sequences that distinguish them from the other secretins, including the well-studied PulD from *Klebsiella* and the GspD secretins from other *E. coli* pathotypes (Figure 5B).

In *E. coli* there is a perfect correlation between the secretin S-domains and the pilotins that are encoded in their genomes: each of the *E. coli* genomes which encoded a secretin in the *Vibrio*-type cluster, also encoded AspS; each of the *E. coli* genomes which encoded a secretin in the *Klebsiella*-type cluster, also encoded a PulS-OutS family member. These protein sequence occurrences are documented in Table S2 and Table S3. Furthermore, the phylogeny of the *Vibrio*-type secretins is reflected in the phylogenetic relationships for the AspS sequences (Figure S2), with one group comprising *E. coli* and close relatives, a slightly more dispersed group of the sequences derived from *Vibrio* sp., and the other genera (*Grimontia* and *Hamiltonella*) more distantly related with respect to the species of *Escherichia* and *Vibrio*.

To test whether the delivery of GspD to the outer membrane by AspS is dependent on this S-domain, we constructed a pETDuet plasmid in which the AspS pilotin and a truncated form of GspD (GspDΔS) lacking the S-domain were co-expressed. Fractionation of membranes from *E. coli* str. BL21(DE3) expressing pETDuet(GspD-C_4_+AspS) or pETDuet (GspDΔS-C_4_+AspS) showed that the pilotin function of AspS depends on the presence of the S-domain (Figure 5C), since GspDΔS is not delivered to the outer membrane. In order to test for a direct recognition event between AspS and the S-domain of *Vibrio*-type secretins, we made use of an assay system previously established for the study of PulD and PulS from *K. oxytoca* [17], [18]. The S-domain sequence from GspD was fused to the maltose-binding protein MalE (MBP-S) and the fusion protein expressed in *E. coli* str. Rosetta(DE3). A His_6_-tagged version of AspS from ETEC was expressed separately. The two proteins were co-purified on Ni-NTA resin. Size-exclusion chromatography of the complex shows co-migration of the pilotin AspS and MBP-S-domain fusion (Figure 5D). Taken together, our data indicate that AspS is required for efficient targeting of GspD to the outer membrane and that the interaction site for AspS is located within the S-domain of the secretin.

### The structure of AspS distinguishes it from the PulS-OutS family of proteins

The AspS pilotin from EPEC (residues 2-112, numbered from the acylated Cys^1^) and *V. cholerae* (residues 6-114) were expressed and purified in soluble form from the periplasm of *E. coli* str. Rosetta(DE3) (see Methods). AspS from EPEC failed to produce well-ordered crystals, whereas *V. cholerae* AspS yielded crystals diffracting to high resolution (Table S4). The structure of *V. cholerae* AspS was solved to 1.48 Å by single wavelength anomalous diffraction method utilizing signal from Zn^2+^ ions present in the crystal.

**Figure 6:**
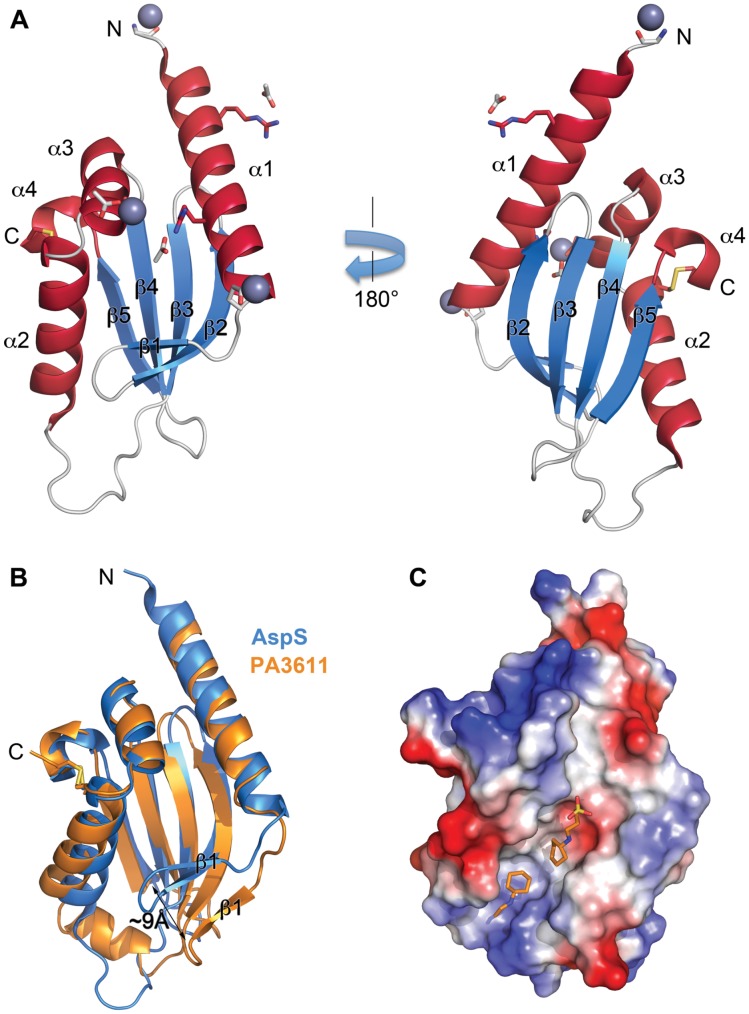
Structure of the pilotin AspS. (A) Ribbon representation of the structure of *V. cholerae* AspS. α-helices, α1–α4, are in crimson; β-strands, β1–β5, are in light blue. Zn^2+^ ions are shown as grey spheres. Acetate ions are shown in stick representation. Residues coordinating Zn^2+^ and acetate ions are in stick representation with oxygen and nitrogen atoms color-coded red and blue, respectively. The position of the disulphide bond Cys74–Cys111 is shown. (B) A superposition of AspS (light blue) and PA3611 (orange) structures in ribbon representation. Note the outward movement of β-strands β1 and β2 in PA3611 structure. (C) Electrostatic surface potential of PA3611 structure (positive = blue; negative = red). The buffer CAPS molecules are shown in stick representation.

The AspS structure is an α/β domain that consists of a 5-stranded β-sheet flanked by 4 α-helices (Figure 6A). The N-terminal helix α1 is followed by antiparallel β-strands β1, β2 and β3. The helices α2 and α3 are arranged across β-strands β4 and β5, which are followed by the C-terminal helix α4. Two conserved cysteine residues, Cys^74^ and Cys^111^, form a disulfide bond that stabilizes the orientation of helix α4 relative to helix α2. The structure of AspS is distinct from the four-helix bundle structures of the previously characterized T2SS pilotins of PulS-OutS family [18], [22]. Moreover, the structure of AspS is different from pilotins of the type III secretion system and the type IV pilus biogenesis system (Supplementary Figure S3). Both DALI and PDBeFold servers identified *P. aeruginosa* protein PA3611 as the closest structural homolog to AspS with an RMSD of 2.1 Å for Cα atoms and 18% sequence identity for 96 aligned residues (Figure 6B) [36], [37] (PDB 3NPD, Joint Center for Structural Genomics, unpublished data). Mapping of sequence conservation across the structure using the ConSurf server [38] showed no obvious conserved surfaces, but did reveal that the disulfide bond is conserved in the various AspS homologs and also in the PA3611 structure from *P. aeruginosa*. The PA3611 structure features an extra α helix after β-strand β3. Also, β-strands β1 and β2 are located closer to helix α2 in AspS compared to PA3611. This open conformation of β-strands β1 and β2 leads to formation of a hydrophobic groove on the surface of PA3611 (Figure 6C). An outward movement of β-strands β1 and β2 in AspS will expose a similar, largely hydrophobic, crevice on the protein surface. We suggest that this region of AspS is involved in interactions with the S-domain of secretin.

## Discussion

A “piggyback” model for the targeting of PulD to the outer membrane of *K. oxytoca* has found general credence in the targeting of secretins for T2SS [19]. This system relies on (i) the outer membrane targeting characteristics of a small lipoprotein, the pilotin, which will be recognized and ferried to the outer membrane by the general lipoprotein targeting “Lol pathway” and (ii) a selective and tight binding of the S-domain of the secretin by the pilotin prior to leaving the inner membrane surface. It has been generally assumed that in the case of T2SS secretins only members of the PulS-OutS family of proteins function in the role of pilotins, and in organisms like *V. cholerae* where no obvious PulS-OutS proteins could be found, targeting of secretins had been thought to be pilotin-independent and mediated by other factors, such as GspA and GspB using functionally-distinct mechanisms [39]. We have clarified this apparent discrepancy by showing that there are at least two classes of T2SS secretins, each having distinguishing targeting sequences and each being targeted by distinct families of pilotin proteins: either the PulS-OutS family or the AspS family.

### A new class of pilotins: the AspS protein family

In the case of the PulS-OutS pilotins, an induced-fit mechanism has been proposed to explain how the natively-disordered S-domain of PulD can be selected for specific and tight binding by the pilotin [17], [18], [22]. Prediction of secondary structure, conserved domains and regions of native-disorder suggest a broadly similar structure for the secretins of the *Klebsiella*-type, as represented by PulD from *K. oxytoca*, and the *Vibrio*-type secretins (Figure S4). Consistent with this and the functional data showing the binding of AspS to the S-domain of *Vibrio*-type secretins, the structure of AspS revealed a candidate binding site for the S-domain peptide. A full structural analysis of the ligand-pilotin complexes involving the PulS-OutS and the AspS pilotins from various species is warranted in order to determine the extent to which the different classes of pilotins select their distinct secretin targets by a conserved mechanism.

During the review of our manuscript, Strozen et al. [35] published a report on YghG demonstrating that it is a lipoprotein located in the outer membrane of enterotoxigenic *E. coli* (ETEC) str. H10407, and showing that deliberate mis-targeting of YghG to the inner membrane resulted in a loss of steady-state levels of GspD in this strain of ETEC. Our kinetic investigation of the assembly of GspD in EPEC directly demonstrates that AspS (*i.e.* YghG) is a pilotin for GspD, and is in agreement with the findings of Strozen et al. [35]. However we disagree with the new nomenclature proposed for YghG, namely that YghG should be referred to as “GspSβ” and that EtpO be refered to as “GspSα”. CLANS analysis illustrates that the *E. coli* protein EtpO, encoded on p157 plasmid of EHEC stains, is a member of the PulS-OutS family and can justifiably be referred to with a generalized “GspS” name. However, AspS is structurally distinct from the PulS-OutS family of proteins. There is a major disadvantage in grouping the two structurally different families (PulS-OutS and AspS) together and applying a single gene-based name (GspS). This would obscure past literature that noted the absence of GspS pilotins in the genomes of *V. cholerae* and other species of bacteria [11], [12], [39]. These important observations from previous studies remain true only as long as the generalized “GspS” name is reserved for pilotins conforming to the conserved domain structure of the PulS-OutS family of proteins.

**Figure 7:**
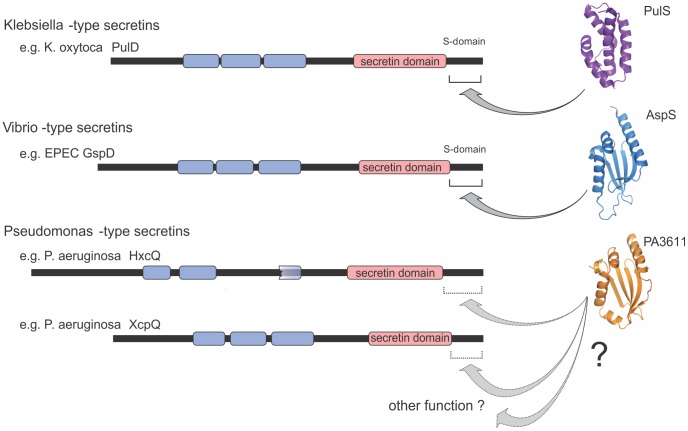
Pilotins distinguished for each class of T2SS secretin. In *K. oxytoca*, PulD has three characterized domains: the N-domains (blue) that dock it to the inner membrane components of the T2SS, the secretin domain (pink) responsible for multimerization, and the C-terminal S-domain, which is critical for PulD to engage the pilotin PulS [14]–[18]. The predicted domain structure of GspD from EPEC is similarly shown, including the S-domain demonstrated to be necessary and sufficient for AspS binding. Also indicated are the T2SS secretins HxcQ and XcpQ from *Pseudomonas*, each of which has a C-terminal extension beyond the recognizable secretin domain which may or may not serve for binding of the protein of unknown function, PA3611.

Previous work on the secretin HxcQ from *Pseudomonas aeruginosa* showed it to be a lipoprotein itself, capable of Lol-dependent targeting to the outer membrane without the assistance of a pilotin [34]. The structure of AspS sheds further light on this scenario, given the structural homology between the AspS pilotins and the protein PA3611 from *P. aeruginosa*. PA3611 is conserved in numerous species of *Pseudomonas* and, while the protein has a signal sequence that would send it into the periplasmic space, there is no signature sequence to suggest that it is a lipoprotein. We suggest that PA3611 might bind to lipoprotein secretins in the periplasm, to stabilize them against proteolysis. Previous studies on other secretins have shown that they can be subject to rapid proteolysis in the periplasm unless protected by the binding of a pilotin [14], [19], [40]. Further study of PA3611 is needed to determine whether it functions as a pilotin or provides some other function in the periplasm of *Pseudomonas* (Figure 7).

### Evolution of T2SS secretins and correlations to substrate selection

There is currently insufficient data from which to trace the evolution of the different types of T2SS secretins, but several conclusions can now be made about sequence relationships within the T2SS secretin family. Phylogenetic analysis of the T2SS secretins demonstrate that the T2SS in all sequenced species of *Vibrio* are closely related to each other and to a discrete subset of T2SS secretins found in some pathotypes and strains of *E. coli* and *Shigella*. In all cases, these bacteria have encoded in their genomes: (a) a T2SS with a *Vibrio*-type secretin, (b) an AspS homolog that could function as a pilotin for the secretin and (c) an AcfD/SslE homolog that could function as an effector protein secreted by the T2SS.

A reasonable explanation for the correlation in finding *Vibrio*-type secretins, AspS-type pilotins and AcfD/SslE substrates in so many diverse bacteria would be that some have acquired a complete functional unit of secretin-pilotin-substrate incorporated with the rest of the T2SS operon. An example of such a “self-contained” system was demonstrated for EPEC [27] and a similar gene organization is apparent in the genomes of *S. boydii* (Figure S1B) and other pathotypes of *E. coli*, while the various genes for protein substrates and the pilotin are dispersed from the T2SS operon in *V. cholerae* (Figure S1B) and in other species of *Vibrio*.

There is an accepted notion that species barriers exist to prevent substrates from one T2SS being secreted by the T2SS of another species [8], [12]. In previous work focussed on a dissection of substrate recognition, the T2SS secretin was shown to be a major determinant of specificity in substrate recognition, and systematic analysis of several substrate proteins led to the suggestion that distinct T2SS substrates have differing requirements for a productive interaction with the OutD secretin [41]. However, despite many documented examples where such a species barrier does appear to exist, there are a few reports of success in expressing a substrate protein from one species for secretion by the T2SS of another species. The studies showing cross-species compliance are now of great interest in the light of the two classes of secretins we have described. The heat-labile enterotoxin (LTB) from enterotoxigenic *E. coli* str. H10407 can be secreted by the T2SS in *V. cholerae* and EPEC [23], [42], and mutations that diminish its recognition by the (*Vibrio*-type) GspD in *E. coli* str. H10407 also diminish recognition by the T2SS in *V. cholera* [43]. However, species of *Dickeya*, *Klebsiella*, *Proteus*, *Serratia* and *Xanthomonas* were shown to be incapable of secreting the LTB substrate [42]. An explanation consistent with all of these results would be that only species with *Vibrio*-type secretins can recognize and secrete substrates derived from organisms (like EPEC) that have *Vibrio*-type secretins in their T2SS machinery.

While it is not yet clear what features in the substrate protein serve as the recognition signal for secretion by the T2SS systems, it is apparent that these are not simple N-terminal sequences as is the case for some other protein transport systems [8]. It has been suggested that complex and structure-based, rather than sequence-based, signals could be encoded in surface features of folded T2SS substrate proteins [41], [44]–[48]. Exactly how secretins would recognize these features of their substrates remains a major question, and the finding that there are distinct classes of secretins provides a new framework on which to start to address this question.

## Materials and Methods

### Strains, plasmids, growth conditions and primers

The bacterial strains and plasmids used in this study are listed in Table S1, using the parental strains enteropathogenic *E. coli* E2348/69, *E. coli* BL21 (DE3) (Invitrogen) and Rosetta (DE3) (Novagen). Strains were grown in Luria Broth (LB) or Casamino acid-yeast extract-salts (CAYE) media supplemented with the appropriate antibiotics (ampicillin 100 µg/ml, kanamycin 30 µg/ml or chloramphenicol 12.5 µg/ml).

Bacterial mutants resulting from the deletion of the genes *gspD*, *yacC* or *yghG(aspS)* were constructed in E2348/69 and BL21 by allelic exchange with *gspD*::Cm^r^, *yacC*::Kan^r^, *aspS*::Kan^r^. These knockouts were generated utilising the λ Red recombinase system carried on plasmid pKD46 [49]. When required the Kan^r^ or Cm^r^ genes were removed using the flanking FRT sites and FLP on plasmid pFT-A [50]. Oligonucleotide primer sequences are available on request.

### Secretin assembly assays

E2348/69 and BL21 (DE3) cells transformed with *gspD-C_4_* expressing plasmids were grown in LB to OD_600_ – 0.6 at 37°C prior to induction with arabinose (0.1%) or IPTG (0.1 mM) respectively for 2 hours at 37°C. After 0, 15, 30, 60 and 120 minutes, 1 ml samples were taken and cells were harvested by centrifugation. Cell pellets were resuspended in a non-standard SDS-PAGE lysis buffer (50 mM KH_2_PO_4_ pH 7.8, 400 mM NaCl, 100 mM KCl, 10% glycerol, 1% DDM and 10 mM imidazole) to minimize dissociation of secretin multimers and 15 µl samples were prepared using Lumio detection (Invitrogen) according to the manufacturer's directions. Samples were analysed by 3–14% SDS-PAGE and fluorometry (Typhoon Trio, Argon Blue 488 nm laser, 520 nm BP40 filter).

### Subcellular fractionation

For experiments requiring membrane isolation, cultures were grown in LB to OD_600_ – 1.0 at 37°C and cells were harvested by centrifugation (5000× *g*, 10 min, 4°C), and resuspended in 0.75 M sucrose/10 mM Tris-HCl, pH 7.5. Lysozyme (50 µg/ml), PMSF (2 mM) and 2 volumes of 1.65 M EDTA, pH 7.5, were added sequentially before cells were homogenized with an EmulsiFlex (Avestin Inc.) at 15,000 psi. Membranes were collected by ultracentrifugation (38,000 rpm, 45 minutes, 4°C), washed and resuspended in 25% (w/v) sucrose in 5 mM EDTA, pH 7.5. Total membranes were fractionated on a six-step sucrose gradient (35∶40∶45∶50∶55∶60% (w/v) sucrose in 5 mM EDTA, pH 7.5) by ultracentrifugation in a SW40 Ti rotor (34,000 rpm, 17 hours, 4°C) and 1 ml fractions were stored at −80°C. 15 µl aliquots of each fraction were prepared using Lumio detection according to the manufacturer's directions and loaded onto a 3–14% SDS-PAGE and analysed by fluorometry and immunoblotting for BamA (serum dilution 1∶2500; [51] and F_1_β serum dilution 1∶8000; [52]).

### Secretome analysis

Cultures were grown in 30 ml of CAYE media for 4 hours. Culture supernatant were isolated and passed through a 0.45 µm filter before the addition of trichloroacetic acid (10% final concentration) and incubated on ice for 1 hour. Precipitated proteins were collected by centrifugation (15,000 rpm, 30 minutes, 4°C) and protein pellets were washed twice with cold 100% methanol. Pellets were allowed to dry and resuspended in SDS sample buffer and 200 µg of protein were loaded onto 3–14% gradient gels for analysis by SDS-PAGE and Coomassie blue R-250 staining.

### Sequence analysis predictions

Lipoprotein signal peptides were predicted with LipoP 1.0 [28], (www.cbs.dtu.dk/services/LipoP), the conserved domain architecture tool CDART [26], (http://www.ncbi.nlm.nih.gov/Structure/lexington/lexington.cgi) was used to define conserved domain boundaries, DISOPRED2 was used to calculate probability of intrinsic disorder [53], (http://bioinf.cs.ucl.ac.uk/index.php?id=806).

Hidden Markov profiles were generated and HMMER searches performed using HMMER v.2.4 [54] to search for the pilotin candidate in *E. coli* and v.3.0 [55] to search for AspS or GspD homologs in *Aeromonas* spp. and *T. auensis*. To find either AspS or YacC related protein sequences, HMMER profiles were generated based on a set of full-length PulS-OutS sequences, the HMMER profile for the PulS_OutS Pfam domain PF09691 available for download from the Pfam website [56], as well as full-length AspS sequences. The HMMER searches were performed against the genomes of *Aeromonas hydrophila* subsp. *hydrophila* ATCC 7966, *Aeromonas veronii* B565 and *Tolumonas auensis* DSM 9187 obtained from the RefSeq database. No hits showed an e-value more significant than 0.1.

### Phylogenetic analyses

Muscle v3.8.31 with the default settings was used for protein sequence alignments [57]. Conserved sites for phylogenetic tree construction were selected by Gblocks [58] under the default settings as implemented in SeaView v.4 [59]. Phylogenetic tree construction was performed with PhyML v3.0 [60] with 500 bootstrap calculations and tree topology searches were performed with the combination of NNI and SPR. Alignment representation for Figure 5A was generated using the JalView version 11 [61] software package, and conservation of the respective amino acids in the alignment is indicated by colours with a cutoff of 40% conservation as implemented in JalView.

### Sequence cluster visualization

Similarity-based clustering analyses were performed using the CLANS software [29], a graph-based sequence similarity visualization software based on sequence similarities obtained by BlastP p-values using BLAST 2.2.26 [62] with default settings as implemented in the CLANS software.

### Cloning, expression and purification of AspS

The gene fragments corresponding to *E. coli* AspS (residues 2-112) and *V. cholerae* AspS (residues 6-114) were cloned into a modified pET-22b(+) vector (Novagen) to encode a periplasmic signal sequence and His_6_ tag followed by a Tobacco Etch Virus (TEV) protease cleavage site. The proteins were expressed in Rosetta(DE3) cells (Novagen) for 3 h at 37°C after induction with 0.5 mM IPTG. Cells were harvested by centrifugation and resuspended in buffer containing 20 mM Tris-HCl pH 8.4, 300 mM NaCl, and 20 mM imidazole. The resuspended cells were lysed using EmulsiFlex-C5 (Avestin) and proteins were purified via a Ni-NTA column (Qiagen). Following the cleavage of His_6_ tag by TEV protease, proteins were purified on size-exclusion Superdex200 column (GE Healthcare) in buffer containing 20 mM HEPES pH 7.5, 100 mM NaCl. Control experiments demonstrated no overlap in the elution profiles of AspS and maltose-binding protein (Figure S5).

### Crystallization and data collection

The crystals of *V. cholerae* AspS were obtained using JCSG Core Suites (Qiagen) [63]. Rod-shaped crystals were grown using vapour diffusion method with 0.2 M Zn acetate, 20% (w/v) PEG 3350 as precipitant. Crystals were briefly soaked in crystallization solution supplemented with 10% (w/v) glycerol and flash-frozen in liquid nitrogen. Data were collected at Southeast Regional Collaborative Access Team (SER-CAT) 22-ID beamline at the Advanced Photon Source, Argonne National Laboratory. The crystals belonged to space group *P*2_1_2_1_2 with one monomer in the asymmetric unit.

### Structure determination and refinement

The AspS structure was solved by single wavelength anomalous diffraction method utilizing signal from Zn^2+^ ions present in the crystal lattice. The Zn^2+^ ion positions were determined using SHELXD and the phases were calculated using autoSHARP [64], [65]. After density modification with SOLOMON as implemented in autoSHARP, the initial model was built using ARP/wARP [66], [67]. The structure was completed using Coot and refined with REFMAC using translation, libration and screw-rotation displacement (TLS) groups as defined by TLSMD server [68]–[70]. The quality of final model was assessed using Molprobity [71]. The structural figures were generated using PyMol (www.pymol.org).

### Accession codes

The coordinates and structure factors for *V. cholerae* AspS were deposited to the Protein Data Bank with accession code 4FTF.

## Supporting Information

Figure S1 **Gene detection, characteristics and synteny for the T2SS in EPEC,** ***Shigella*** **and** ***Vibrio cholerae*** **.**
(PDF)

Figure S2 **Phylogenetic relationships for the AspS family of proteins.**
(PDF)

Figure S3 **Structures of pilotins.**
(PDF)

Figure S4 **Domain structure and disorder predictions for GspD and PulD.**
(PDF)

Figure S5 **The S-domain of secretin mediates binding of AspS.**
(PDF)

Table S1 **Strains and plasmids used in this study.**
(PDF)

Table S2 **Secretin accession numbers.**
(PDF)

Table S3 **PulS-OutS, YacC and AspS accession numbers.**
(PDF)

Table S4 **Data collection and refinement statistics.**
(PDF)
