# Supplementary material for: Assembly of the Type II Secretion System such as Found in Vibrio cholerae Depends on the Novel Pilotin AspS
Source: PLoS Pathog. 2013 Jan 10;9(1):e1003117. doi: 10.1371/journal.ppat.1003117 (PMC3542185; doi:10.1371/journal.ppat.1003117)
Supplement: Figure S2 — Phylogenetic relationships for the AspS family of proteins. (PDF) [file ppat.1003117.s002.pdf]

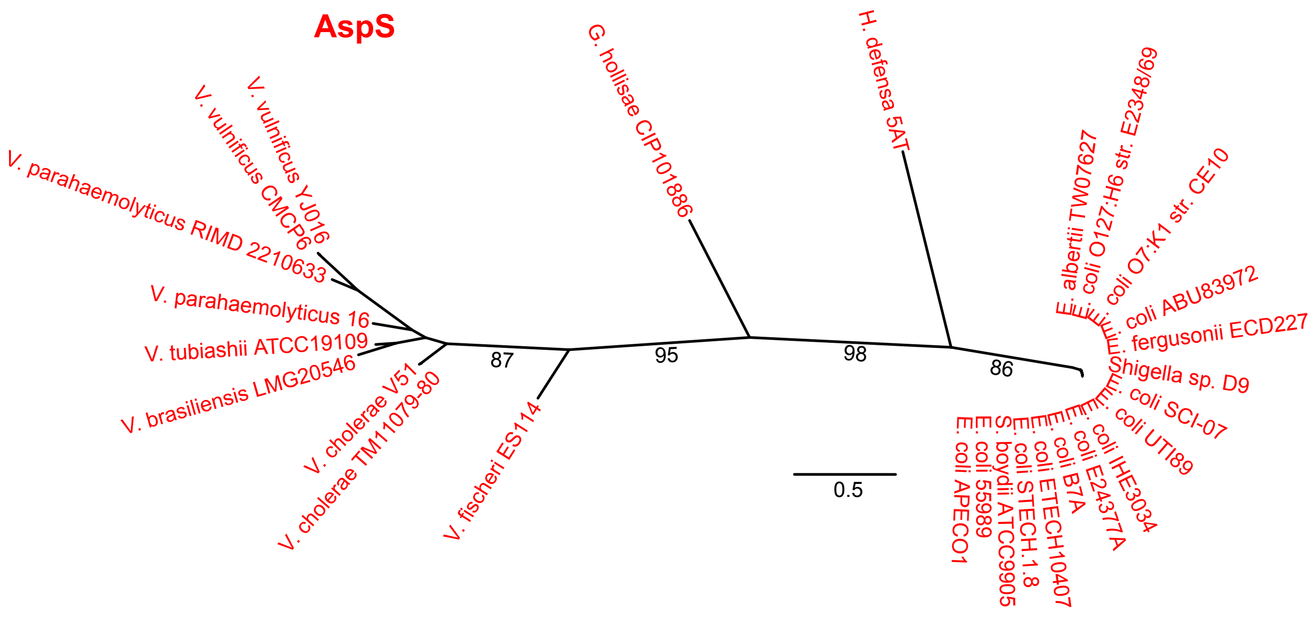

Supplementary Figure S2. Phylogenetic relationships for the AspS family of proteins. Phylogenetic analysis of AspS sequences mirrors the relationships observed for Vibrio-type secretins. Phylogenetic tree reconstruction was performed with PhyML v3.0 [Guindon et al. 2003] using 500 bootstrap calculations and shown as percentage values, further details are described in the Methods. For a full list of the sequence accession numbers see Table S3.

Reference

Guindon S, Gascuel O (2003) A simple, fast, and accurate algorithm to estimate large phylogenies by maximum likelihood. *Syst Biol* **52:**696-704.
