# Supplementary material for: Assembly of the Type II Secretion System such as Found in Vibrio cholerae Depends on the Novel Pilotin AspS
Source: PLoS Pathog. 2013 Jan 10;9(1):e1003117. doi: 10.1371/journal.ppat.1003117 (PMC3542185; doi:10.1371/journal.ppat.1003117)
Supplement: Figure S3 — Structures of pilotins. (PDF) [file ppat.1003117.s003.pdf]

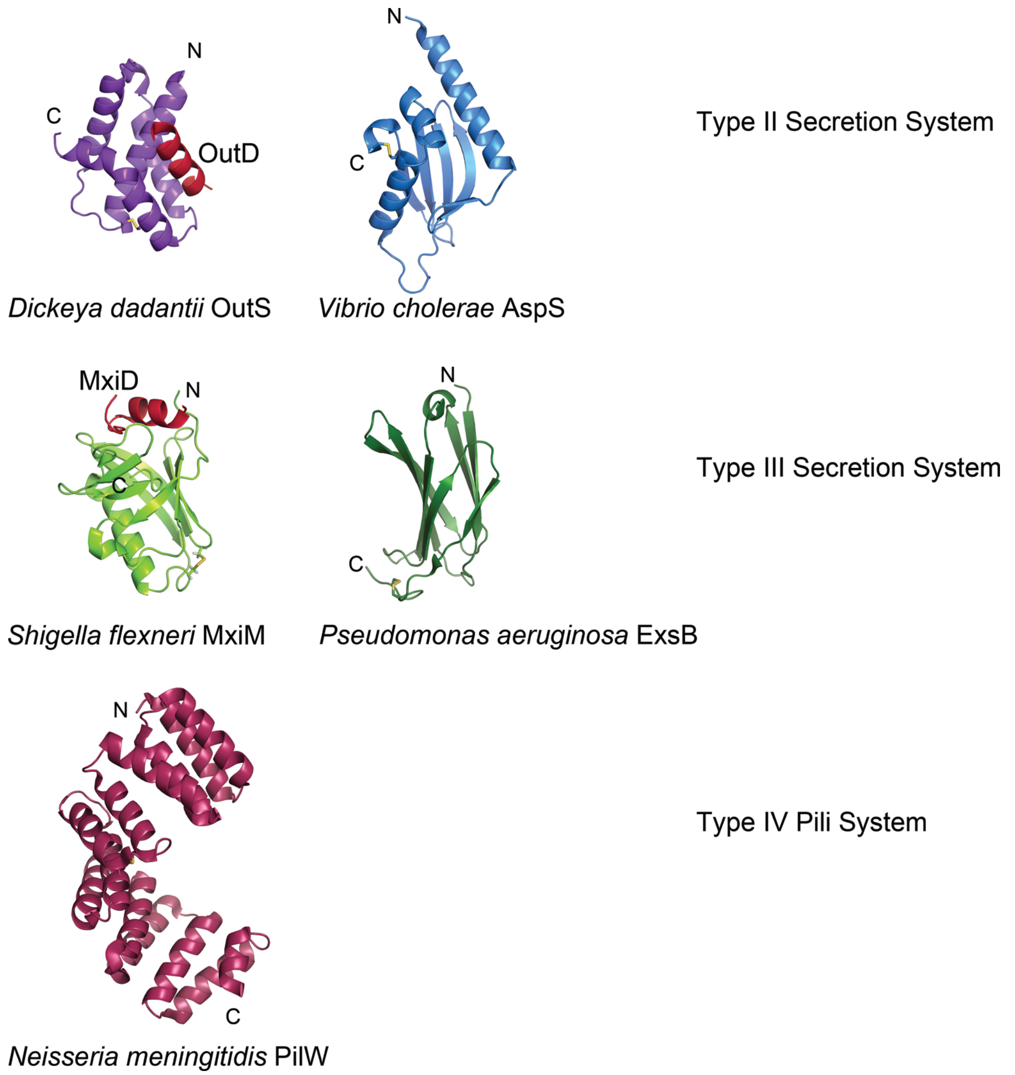

Supplementary Figure S3. Structures of pilotins.
The available structures of pilotins display different folds, albeit they have similar functions. The structure of *D. dadantii* OutS in complex with the secretin fragment OutD (Gu *et al* 2012). Homologous structures of *Klebsiella oxytoca* PulS and enterohemorrhagic *E. coli* GspS are available (Tosi *et al* 2011; Korotkov & Hol 2011). The structure of *V. cholerae* AspS (this work). The structure of *S. flexneri* pilotin MxiM in complex with the secretin fragment MxiD (Okon *et al* 2008). The structure of *P. aeruginosa* pilotin ExsB (Izoré *et al* 2011). The structure of *N. meningitidis* pilotin PilW (Trindade *et al* 2008). Homologous structures of *P. aeruginosa* pilotin PilF are available (Kim *et al* 2006; Koo *et al* 2008).

References
Gu S, Rehman S, Wang X, Shevchik VE, Pickersgill RW (2012) Structural and functional insights into the pilotin-secretin complex of the type II secretion system. *PLoS Pathogens* **8:** e1002531
Izoré T, Perdu C, Job V, Attree I, Faudry E, Dessen A. (2011) Structural characterization and membrane localization of ExsB from the type III secretion system (T3SS) of

Pseudomonas aeruginosa. *J. Mol. Biol.* **413:** 236-246
Kim K, Oh J, Han D, Kim EE, Lee B, Kim Y. (2006) Crystal structure of PilF: functional implication in the type 4 pilus biogenesis in *Pseudomonas aeruginosa*. *Biochem. Biophys. Res. Commun.* **340:** 1028-1038
Koo J, Tammam S, Ku SY, Sampaleanu LM, Burrows LL, Howell PL. (2008) PilF is an outer membrane lipoprotein required for multimerization and localization of the *Pseudomonas aeruginosa* Type IV pilus secretin. *J. Bacteriol.* **190:** 6961-6969
Korotkov KV, Hol WGJ (2011) Crystal structure of the type 2 secretion system pilotin GspS. DOI:10.2210/pdb3sol/pdb
Okon M, Moraes TF, Lario PI, Creagh AL, Haynes CA, Strynadka NC, McIntosh LP (2008) Structural characterization of the type-III pilot-secretin complex from *Shigella flexneri*. *Structure* **16:** 1544-1554
Tosi T, Nickerson NN, Mollica L, Jensen MR, Blackledge M, Baron B, England P, Pugsley AP, Dessen A (2011) Pilotin-secretin recognition in the type II secretion system of *Klebsiella oxytoca*. *Mol. Microbiol.* **82:** 1422-1432
Trindade MB, Job V, Contreras-Martel C, Pelicic V, Dessen A. (2008) Structure of a widely conserved type IV pilus biogenesis factor that affects the stability of secretin multimers. *J. Mol. Biol.* **378:** 1031-1039
