# Supplementary material for: Assembly of the Type II Secretion System such as Found in Vibrio cholerae Depends on the Novel Pilotin AspS
Source: PLoS Pathog. 2013 Jan 10;9(1):e1003117. doi: 10.1371/journal.ppat.1003117 (PMC3542185; doi:10.1371/journal.ppat.1003117)
Supplement: Figure S4 — Domain structure and disorder predictions for GspD and PulD. (PDF) [file ppat.1003117.s004.pdf]

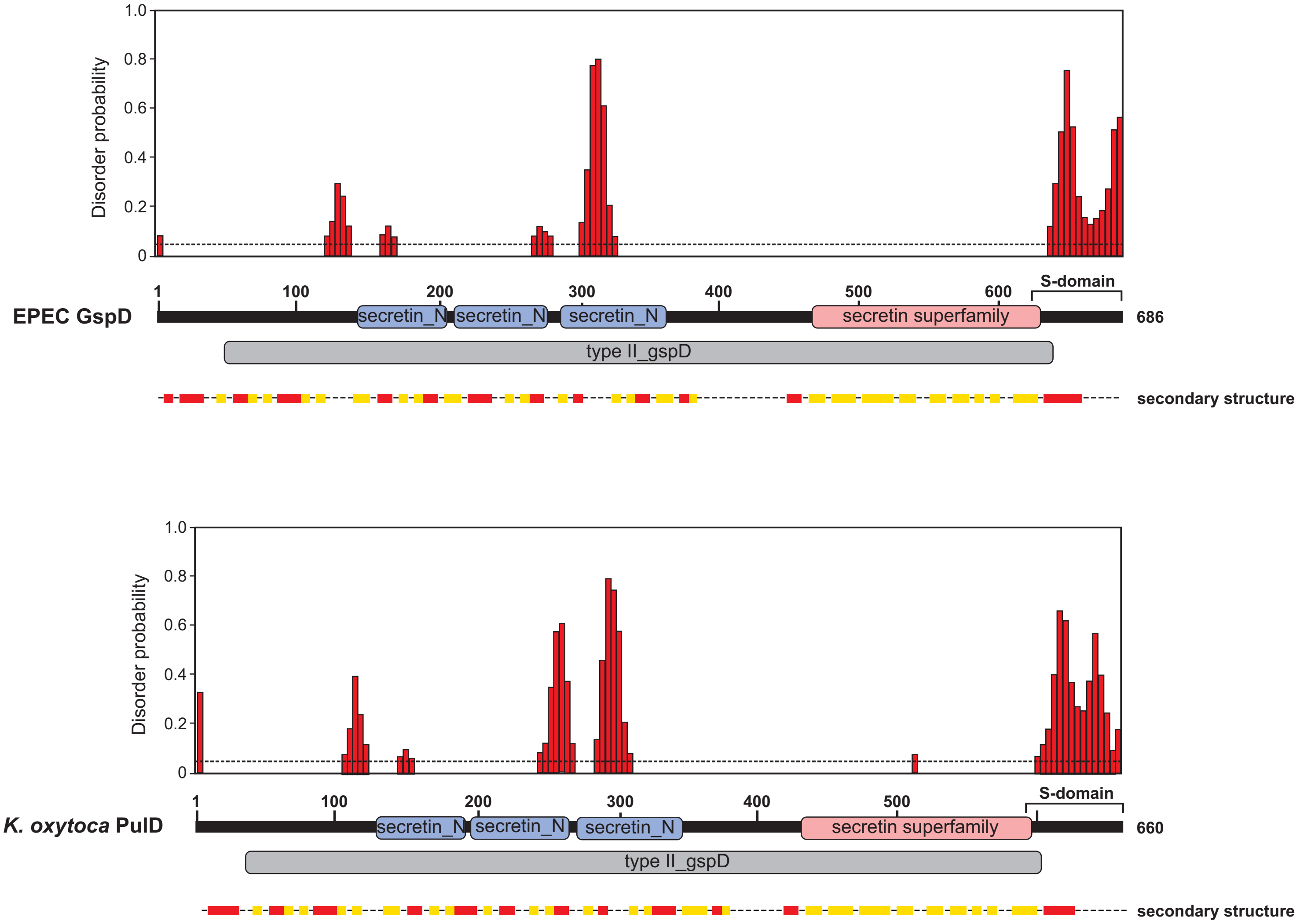

Supplementary Figure S4. Domain structure and disorder predictions for GspD and PulD. The sequences of GspD from EPEC (YP_002330719.1) and PulD from *K. oxytoca* (EHT13409.1) were analysed by CDART to position the conserved domain boundaries and DISOPRED2 to calculate probability of intrinsic disorder (Ward et al. 2004). The dotted line represents the 5% probability cut-off and all values greater than this are plotted with red bars. The DISOPRED2 calculations are aligned to scale with the amino acid residue numbers in the domain representation of each protein. The intrinsic disorder in the S-domain of PulD has been experimentally verified (Nickerson *et al* 2011; Tosi *et al* 2011; Gu *et al* 2012) and the intrinsic disorder prediction for EPEC GspD is equivalent to that for PulD. Secondary structure predictions were calculated using JPred (Cole *et al.* 2008), red = α-helix, yellow = β-strand.

References

Cole C, Barber JD & Barton GJ. (2008) *Nucleic Acids Res.* **35** (suppl. 2) W197-W201

Gu S, Rehman S, Wang X, Shevchik VE, Pickersgill RW (2012) Structural and functional insights into the pilotin-secretin complex of the type II secretion system. *PLoS Pathogens* **8:** e1002531

Nickerson NN, Tosi T, Dessen A, Baron B, Raynal B, England P, Pugsley AP (2011) Outer membrane targeting of secretin PulD protein relies on disordered domain recognition by a dedicated chaperone. *J. Biol. Chem.* **286:** 38833-38843

Tosi T, Nickerson NN, Mollica L, Jensen MR, Blackledge M, Baron B, England P, Pugsley AP, Dessen A (2011) Pilotin-secretin recognition in the type II secretion system of *Klebsiella oxytoca*. *Mol. Micro.* **82:** 1422-1432

Ward JJ, Sodhi JS, McGuffin LJ, Buxton BF, Jones DT (2004) Prediction and functional analysis of native
