# Supplementary material for: Assembly of the Type II Secretion System such as Found in Vibrio cholerae Depends on the Novel Pilotin AspS
Source: PLoS Pathog. 2013 Jan 10;9(1):e1003117. doi: 10.1371/journal.ppat.1003117 (PMC3542185; doi:10.1371/journal.ppat.1003117)
Supplement: Figure S5 — The S-domain of secretin mediates binding of AspS. (PDF) [file ppat.1003117.s005.pdf]

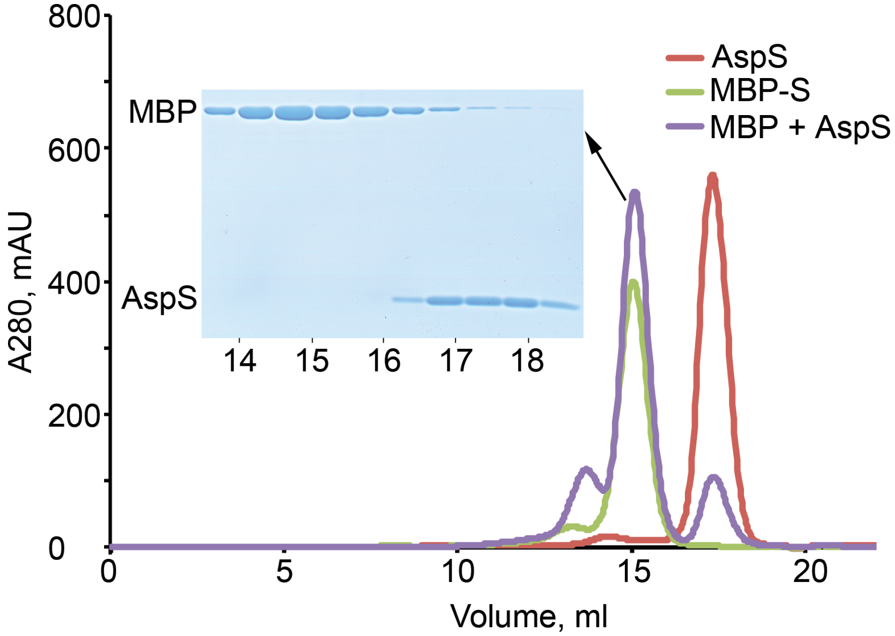

Supplementary Figure S5: The S-domain of secretin mediates binding of AspS. This is a control experiment to the results shown in Figure 5D. Size-exclusion chromatography profiles of the purified AspS (red), the purified MBP-S-domain fusion (green), and the mixture of AspS and MBP (purple) on a Superdex200 column. An SDS-PAGE gel of the fractions of the mixture of AspS and MBP shows an apparent lack of complex formation, which confirms that the AspS binding is mediated by the S-domain of secretin. A280, absorbance at 280 nm; mAU, milli absorbance units.
