# Supplementary material for: Assembly of the Type II Secretion System such as Found in Vibrio cholerae Depends on the Novel Pilotin AspS
Source: PLoS Pathog. 2013 Jan 10;9(1):e1003117. doi: 10.1371/journal.ppat.1003117 (PMC3542185; doi:10.1371/journal.ppat.1003117)
Supplement: Table S1 — Strains and plasmids used in this study. (PDF) [file ppat.1003117.s006.pdf]

Supplementary Table S1: Strains and plasmids used in this study.

	**Description**	**Reference**
**Strain**		
E2348/69	EPEC serotype O127:H6	Levine et al 1978
E2348/69 *ΔgspD*	E2348/69 *ΔgspD*	Baldi et al 2012
E2348/69 *ΔsslE*	E2348/69 *ΔsslE*	Baldi et al 2012
E2348/69 *ΔyacC*	E2348/69 *ΔyacC*	This Study
E2348/69 *ΔaspS*	E2348/69 *ΔaspS*	This Study
E2348/69 *ΔgspD ΔyacC*	E2348/69 *ΔgspD ΔyacC*	This Study
E2348/69 *ΔgspD ΔaspS*	E2348/69 *ΔgspD*::Cm^r, *ΔaspS*	This Study
BL21 (DE3)	*E. coli* strain: F- *ompT hsdS*B (rB- mB-) *dcm gal* (DE3)	Invitrogen
BL21 (DE3) *ΔgspD ΔaspS*	BL21 (DE3) *ΔgspD*::Cm^r, *ΔaspS*	This Study
Rosetta (DE3)	*E. coli* strain: F- *ompT hsdS*B (rB- mB-) *dcm gal* (DE3)	Novagen
**Plasmid**		
pBAD24	*ori* pMB1, Amp^r	Guzman et al 1995
pET DUET-1	*ori ColE1*, *lacI* gene, Amp^r , used for assembly assays in BL21(DE3)	Novagen
pET DUET-1 GspD-C_4	E2348/69 *gspD-C_4* was cloned into NcoI/HindIII sites of pET DUET-1	This Study
pET DUET-1 GspD-C_4 YacC	E2348/69 *yacC* was cloned into NdeI/XhoI sites of pET DUET-1 GspD-C_4	This Study
pET DUET-1 GspD-C_4 AspS	E2348/69 *aspS* was cloned into NdeI/XhoI sites of pET DUET-1 GspD-C_4	This Study
pFT-A	*ori* R101, *repA*101ts, flp, Amp^r	Posfai et al 1997
pGEM-T-Easy	*ori* pMB1, Amp^r	Promega
pKD3	FRT flanked Cm^r gene, Cm^r, Amp^r	Datsenko and Wanner, 2000

pKD4	FRT flanked Kan^r gene, Kan^r, Amp^r	Datsenko and Wanner, 2000
pKD46	λ Red recombinase, *ori* R101, *repA*101ts, Amp^r	Datsenko and Wanner, 2000
pJP117	E2348/69 *gspD-C$_4$* is cloned into the *Nco*I/*Xba*I sites of pNM12 (pBAD24 modified to include MscI site). Used for assembly assays in E2348/69	This Study
pJP168	P_{BAD} promoter and *araC* replaced by P_{tetA} and *tetR*	Baldi et al 2012
pJP181	E2348/69 *gspD-C$_4$* is cloned into the *Nco*I/*Hind*III sites of pJP168. Used for sucrose fractionation in E2348/69.	This Study
pKV1066	H10407 *yghG* was cloned into a modified pET-22b(+) vector (Novagen) to encode *pelB* periplasmic signal sequence, His$_6$ tag and tobacco etch virus protease cleavage site; *ori ColE1*, *lacI* gene, Amp^r; used for binding assay with the secretin S-domain	This study
pKV1111	Sequence corresponding to 60 C-terminal residues of H10407 *gspD* (S-domain) was cloned into a pET-22b(+) vector to encode a fusion with *malE* gene; *ori ColE1*, *lacI* gene, Amp^r; used for binding assay with the pilotin	This study
pKV1112	VC395_1831 from *V. cholerae* 569B (corresponds to VC1703 from *V. cholerae* O1 biovar El Tor str. N16961) was cloned into a modified pET-22b(+) vector to encode *pelB* periplasmic signal sequence, His$_6$ tag and tobacco etch virus protease cleavage site; *ori ColE1*, *lacI* gene, Amp^r	This study

REFERENCES

Baldi DL, Higginson EE, Hocking DM, Praszkier J, Cavaliere R et al. (2012) The Type II Secretion System and Its Ubiquitous Lipoprotein Substrate, SslE, Are Required for Biofilm Formation and Virulence of Enteropathogenic *Escherichia coli*. Infect Immun 80: 2042-2052

Guzman LM, Belin D, Carson MJ, Beckwith J (1995) Tight regulation, modulation, and high-level expression by vectors containing the arabinose PBAD promoter. J Bacteriol 177: 4121-4130

Levine MM, Bergquist EJ, Nalin DR, Waterman DH, Hornick RB, et al. (1978) *Escherichia coli* strains that cause diarrhoea but do not produce heat-labile or heat-stable enterotoxins and are non-invasive. Lancet 1: 1119-1122

Posfai G, Koob MD, Kirkpatrick HA, Blattner FR (1997) Versatile insertion plasmids for targeted genome manipulations in bacteria: isolation, deletion, and rescue of the pathogenicity island LEE of the *Escherichia coli* O157:H7 genome. J Bacteriol 179: 4426-4428

Datsenko KA, Wanner BL (2000) One-step inactivation of chromosomal genes in *Escherichia coli* K-12 using PCR products. Proc. Natl. Acad. Sci. U.S.A. 97: 6640-6645
