# Supplementary material for: Assembly of the Type II Secretion System such as Found in Vibrio cholerae Depends on the Novel Pilotin AspS
Source: PLoS Pathog. 2013 Jan 10;9(1):e1003117. doi: 10.1371/journal.ppat.1003117 (PMC3542185; doi:10.1371/journal.ppat.1003117)
Supplement: Table S2 — Secretin accession numbers. (PDF) [file ppat.1003117.s007.pdf]

Supplementary Table S2: Secretin accession numbers.

Species-strain	T2SS secretin
Aeromonas hydrophila (ExeD)	CAA47124.1
Aeromonas hydrophila subsp. hydrophila ATCC 7966 (ExeD)	YP_855102.1
Aeromonas veronii B565 (ExeD)	YP_004393542.1
Candidatus Hamiltonella defensa 5AT (Acyrthosiphon pisum)	YP_002923157.1
Citrobacter koseri ATCC BAA-895 (GspD)	YP_001453787.1
Citrobacter rodentium ICC168 (GspD)	YP_003367904.1
Dickeya dadantii 3937 (SttD)	YP_003883459.1
Dickeya dadantii 3937 (OutD)	YP_003883933.1
Dickeya zeae Ech1591 (SttD)	YP_003005612.1
Dickeya zeae Ech1591 (OutD)	YP_003003641.1
Enterobacter cancerogenus ATCC 35316 (GspD)	ZP_05967041.1
Enterobacter cloacae SCF1 (GspD)	YP_003942468.1
Erwinia sp. Ejp617 (OutD)	ADP10010.1
Escherichia albertii TW07627 (GspD)	ZP_02900292.1
Escherichia coli 55989 (GspD)	YP_002404340.1
Escherichia coli ABU 83972 (Klebsiella-type GspD)	YP_006107718.1
Escherichia coli ABU 83972 (GspD)	YP_006107361.1
Escherichia coli APEC O1 (Klebsiella-type GspD)	YP_858926.1
Escherichia coli APEC O1 (GspD)	YP_854407.1
Escherichia coli B7A (GspD)	ZP_03030864.1
Escherichia coli E24377A (GspD)	YP_001464424.1
Escherichia coli ETEC H10407 (Klebsiella-type GspD)	YP_006117117.1
Escherichia coli ETEC H10407 (GspD)	YP_006116778.1
Escherichia coli IHE3034 (Klebsiella-type GspD)	YP_006102848.1
Escherichia coli IHE3034 (GspD)	YP_006102496.1
Escherichia coli O127:H6 str. E2348/69 (GspD)	YP_002330719.1
Escherichia coli O157:H7 EDL933 (EptD)	YP_325593.1
Escherichia coli O157:H7 str. EC4045 (EptD)	ZP_03258459.1
Escherichia coli O157:H7 str. EC4115 (EptD)	YP_002268498.1
Escherichia coli O7:K1 str. CE10 (GspD)	YP_006145502.1
Escherichia coli SCI-07 (GspD)	EIA35015.1
Escherichia coli SCI-07 (GspD)	EIA35550.1
Escherichia coli STEC_EH250 (GspD)	EGW92118.1
Escherichia coli STEC_H.1.8 (GspD)	EGX06967.1
Escherichia coli UTI89 (GspD)	YP_542748.1
Escherichia coli UTI89 (GspD)	YP_542363.1
Escherichia fergusonii ECD227 (GspD)	EGC96390.1
Grimontia hollisae CIP 101886 (EpsD)	ZP_06050984.1
Klebsiella oxytoca 10-5246 (PulD)	EHT13409.1
Klebsiella oxytoca 10-5250 (PulD)	EHT07150.1
Klebsiella pneumoniae 342 (PulD)	YP_002240367.1

Legionella longbeachae D-4968 (LspD)	ZP_06185717.1
Legionella pneumophila str. Paris (LspD)	YP_123599.1
Pectobacterium carotovorum subsp. brasiliensis PBR1692 (OutD)	ZP_03827789.1
Pectobacterium wasabiae WPP163 (OutD)	YP_003258818.1
Pseudomonas aeruginosa PAO1 (XcpQ)	CAA48582.1
Pseudomonas aeruginosa PAO1 (HxcQ)	NP_249376.1
Pseudomonas aeruginosa PAO1 (XqhA)	NP_250559.1
Pseudomonas fluorescens F113 (HxcQ)	YP_005209094.1
Pseudomonas fluorescens F113 (XcpQ)	YP_005205867.1
Rahnella aquatilis HX2	YP_005400169.1
Serratia proteamaculans 568	YP_001480473.1
Serratia plymuthica PRI-2C	EIJ17929.1
Shigella boydii ATCC 9905 (GspD)	EFW55099.1
Shigella boydii ATCC 9905 (GspD)	EFW55932.1
Shigella sp. D9 (GspD)	ZP_08392631.1
Tolumonas auensis DSM 9187 (ExeD)	YP_002891583.1
Vibrio brasiliensis LMG 20546 (EpsD)	ZP_08099671.1
Vibrio cholerae TM 11079-80 (EpsD)	ZP_04411225.1
Vibrio cholerae V51 (EpsD)	ZP_04919061.1
Vibrio fischeri ES114 (EpsD)	YP_205857.1
Vibrio parahaemolyticus 16 (EpsD)	ZP_05119391.1
Vibrio parahaemolyticus RIMD 2210633 (EpsD)	NP_796512.1
Vibrio tubiashii ATCC 19109 (EpsD)	ZP_08739448.1
Vibrio vulnificus CMCP6 (EpsD)	NP_759853.1
Vibrio vulnificus YJ016 (EpsD)	NP_933007.1
Xanthomonas campestris pv. vesicatoria str. 85-10 (XpsD)	YP_365389.1
Xanthomonas campestris pv. vesicatoria str. 85-10 (XcsD)	YP_362487.1
Yersinia aldovae ATCC 35236 (YstD)	ZP_04621755.1
Yersinia aldovae ATCC 35236 (YstD)	ZP_04620085.1
Yersinia enterocolitica subsp. enterocolitica 8081 (YstD)	YP_001007521.1
Yersinia enterocolitica subsp. enterocolitica 8081 (YstD)	YP_001007724.1
Yersinia pestis KIM10+ (YstD)	NP_670503.1

Pink denotes those secretins that fall within the “Vibrio-type” shown in Figure 4.
