# Supplementary material for: Assembly of the Type II Secretion System such as Found in Vibrio cholerae Depends on the Novel Pilotin AspS
Source: PLoS Pathog. 2013 Jan 10;9(1):e1003117. doi: 10.1371/journal.ppat.1003117 (PMC3542185; doi:10.1371/journal.ppat.1003117)
Supplement: Table S3 — PulS-OutS, YacC and AspS accession numbers. (PDF) [file ppat.1003117.s008.pdf]

Supplementary Table S3: PulS-OutS, YacC and AspS accession numbers.

Species with *Klebsiella*-type secretins						Species with *Vibrio*-type secretins		
**Species-strain**	**Accession**	**Protein**	**Species-strain**	**Accession**	**Protein**	**Species-strain**	**Accession**	**Protein**
Klebsiella oxytoca 10-5246	EHT13413.1	PulS	Citrobacter koseri ATCC BAA-895	YP_001454765.1	YacC	Candidatus Hamiltonella defensa 5AT	YP_002923249.1	AspS
Klebsiella oxytoca 10-5250	EHT07154.1	PulS	Citrobacter rodentium ICC168	YP_003363779.1	YacC	Escherichia albertii TW07627	ZP_02900670.1	AspS
Klebsiella pneumoniae 342	YP_002240362.1	PulS	Dickeya dadantii 3937	YP_003884188.1	YacC	Escherichia coli 55989	YP_002404342.1	AspS
Dickeya dadantii 3937	ADM99380.1	OutS	Dickeya zeae Ech1591	YP_003003369.1	YacC	Escherichia coli ABU 83972	YP_006107363.1	AspS
Dickeya zeae Ech1591	YP_003003638.1	OutS	Enterobacter cancerogenus ATCC 35316	ZP_05966740.2	YacC	Escherichia coli APEC O1	YP_854410.1	AspS
Pectobacterium wasabiae WPP163	YP_003258814.1	OutS	Enterobacter cloacae SCF1	YP_003943129.1	YacC	Escherichia coli B7A	ZP_03030859.1	AspS
Pectobacterium carotovorum subsp. brasiliensis PBR1692	ZP_03827794.1	OutS	Erwinia sp. Ejp617	YP_005816839.1	YacC	Escherichia coli E24377A	YP_001464426.1	AspS
Escherichia coli O157:H7 EDL933	YP_325604.1	EtpO	Escherichia coli 55989	YP_002401255.1	YacC	Escherichia coli ETEC H10407	YP_006116780.1	AspS
Escherichia coli O157:H7 str. EC4045	ZP_03258481.1	EtpO	Escherichia coli ABU 83972	YP_006104279.1	YacC	Escherichia coli IHE3034	YP_006102498.1	AspS
Escherichia coli O157:H7 str. EC4115	YP_002268509.1	EtpO	Escherichia coli APEC O1	YP_851322.1	YacC	Escherichia coli O127:H6 str. E2348/69	YP_002330721.1	AspS

Serratia proteamaculans 568	YP_001479335.1	PulS-OutS family	Escherichia coli B7A	ZP_03028195.1	YacC	Escherichia coli O7:K1 str. CE10	YP_006145504.1	AspS
Serratia plymuthica PRI-2C	ZP_10113051.1	PulS-OutS family	Escherichia coli E24377A	YP_001461290.1	YacC	Escherichia coli SCI-07	EIA35552.1	AspS
Yersinia aldovae ATCC 35236	ZP_04621856.1	YtsS	Escherichia coli ETEC H10407	YP_006113711.1	YacC	Escherichia coli STEC_H.1.8	EGX06969.1	AspS
Yersinia enterocolitica subsp. enterocolitica 8081	YP_001007735.1	YtsS	Escherichia coli IHE3034	YP_006099379.1	YacC	Escherichia coli UTI89	YP_542365.1	AspS
Erwinia sp. Ejp617	YP_005816908.1	OutS	Escherichia coli O127:H6 str. E2348/69	YP_002327717.1	YacC	Escherichia fergusonii ECD227	EGC96388.1	AspS
			Escherichia coli O157:H7 EDL933	NP_285818.1	YacC	Grimontia hollisae CIP 101886	ZP_06053900.1	AspS
			Escherichia coli O157:H7 str. EC4045	ZP_03254912.1	YacC	Shigella boydii ATCC 9905	EFW55930.1	AspS
			Escherichia coli O157:H7 str. EC4115	YP_002268729.1	YacC	Shigella sp. D9	ZP_08392629.1	AspS
			Escherichia coli O7:K1 str. CE10	YP_006142260.1	YacC	Vibrio brasiliensis LMG 20546	ZP_08098413.1	AspS
			Escherichia coli SCI-07	EIA38197.1	YacC	Vibrio cholerae TM 11079-80	ZP_04410118.1	AspS
			Escherichia coli STEC_EH250	EGW99191.1	YacC	Vibrio cholerae V51	ZP_04918467.1	AspS
			Escherichia coli STEC_H.1.8	EGX16937.1	YacC	Vibrio fischeri ES114	YP_204405.1	AspS
			Escherichia coli UTI89	YP_539176.1	YacC	Vibrio parahaemolyticus 16	ZP_05121506.1	AspS
			Escherichia fergusonii	EGC93914.1	YacC	Vibrio parahaemolyticus	NP_798352.1	AspS

			ECD227			RIMD 2210633		
			Klebsiella oxytoca 10-5246	EHT13356.1	YacC	Vibrio tubiashii ATCC 19109	ZP_08739870.1	AspS
			Klebsiella oxytoca 10-5250	EHT07120.1	YacC	Vibrio vulnificus CMCP6	NP_761873.1	AspS
			Klebsiella pneumoniae 342	YP_002240398.1	YacC	Vibrio vulnificus YJ016	NP_934003.1	AspS
			Pectobacterium carotovorum subsp. brasiliensis PBR1692	ZP_03826617.1	YacC			
			Pectobacterium wasabiae WPP163	YP_003258571.1	YacC			
			Rahnella aquatilis HX2	YP_005403530.1	YacC			
			Serratia proteamaculans 568	YP_001480223.1	YacC			
			Serratia plymuthica PRI-2C	ZP_10112392.1	YacC			
			Shigella boydii ATCC 9905	EFW54632.1	YacC			
			Shigella sp. D9	ZP_08393601.1	YacC			
			Yersinia aldovae ATCC 35236	ZP_04621045.1	YacC			
			Yersinia enterocolitica subsp. enterocolitica 8081	YP_001005056.1	YacC			
			Yersinia pestis KIM10+	NP_668112.1	YacC			
