# Supplementary material for: Assembly of the Type II Secretion System such as Found in Vibrio cholerae Depends on the Novel Pilotin AspS
Source: PLoS Pathog. 2013 Jan 10;9(1):e1003117. doi: 10.1371/journal.ppat.1003117 (PMC3542185; doi:10.1371/journal.ppat.1003117)
Supplement: Figure S1 — Gene detection, characteristics and synteny for the T2SS in EPEC, Shigella and Vibrio cholerae . (PDF) [file ppat.1003117.s001.pdf]

A.

```
EPEC        CAS----HNENASLLAKKQAQNISQNLPIKSAGYTLVLAQSSGTTVKMTIISEAGAQTTQTPDAFLTSYQRQMCADPTVKLMITEGINYSITINDTRTGNQYQRKLDRTTCGIVKA
S.boydii    CAS----HNENASLLAKKQAQNISQNLPIKSAGYTLVLAQSSGTTVKMTIISEAGTQTTQTPDAFLTSYQRQMCADPTVKLMITEGINYSITINDTRTGNQYQRKLDRTTCGIVKA
V.cholerae  CSSGSAEKQRNLELLAGNRASLLSTELPLEFGPLNILRATAKGSTVELMMVYNTDANNAKPTEQVLQSAVSSFCANKDIRSNLDVGISYRIQMRNTRGQLMADQLVTKESCKQG
            *:*    ::.* .*** ::*. :* :**:: .   .:: * :.*:**:: :: ::.:::..: .* *    .:**:    :: :   **.* * :.:**      :: : : :*
```

B.

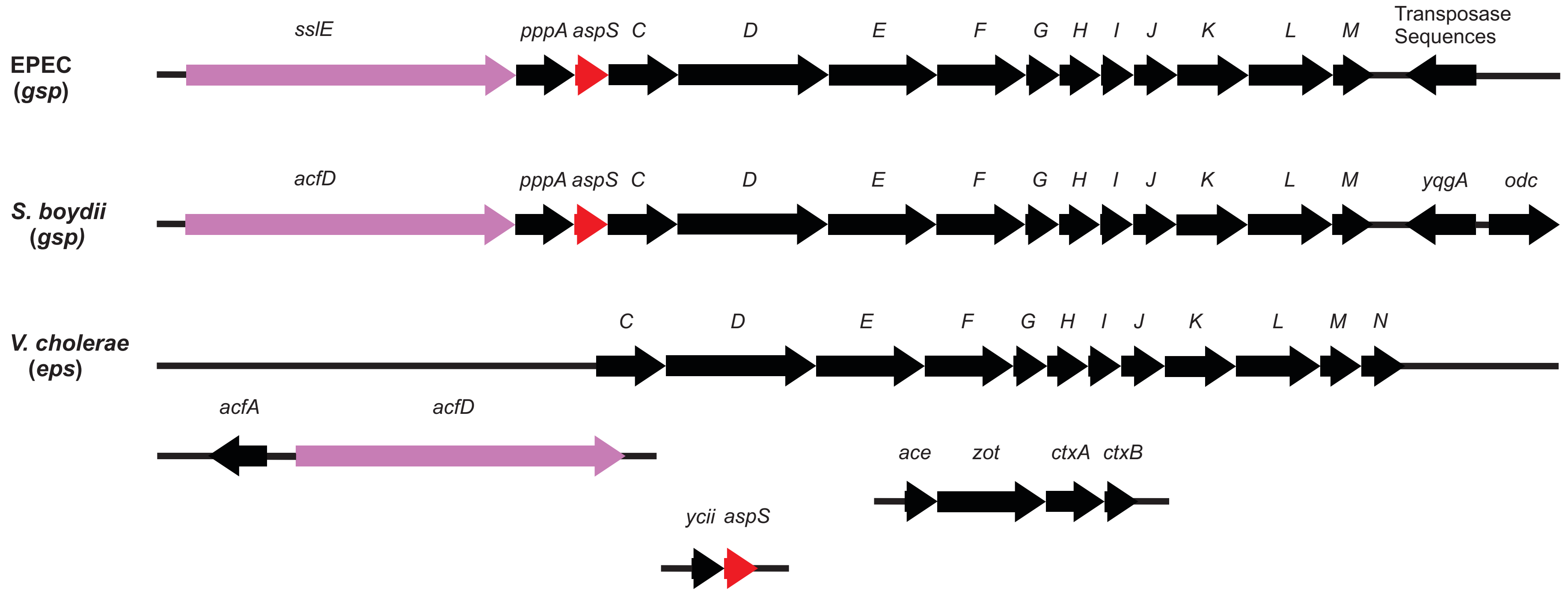

Supplementary Figure S1. Gene detection, characteristics and synteny for the T2SS in EPEC, *Shigella* and *Vibrio cholerae*.

(A) Multiple sequence alignment of AspS homologs, aligned from the cysteine residue predicted to be at +1 and acylated after the signal peptidase cleavage, (*) indicates sequence identity (:) indicates highly conserved (.) indicates some degree of conservation. Disulfide-linked cysteine residues are underlined. (B) The Comprehensive Microbial Resource (CMR) was used to detect sequence relationships in the genomes of EPEC (i.e. enteropathogenic *E. coli* O127:H6 str. E2348/69), *Shigella boydii* D9 and *Vibrio cholerae*. The genes are coloured for the pilotin AspS (red) and the substrate SslE/AcfD (pink), and neighbouring genes are depicted in black and drawn to scale according to their length.
