# Supplementary material for: Assembly of the Type II Secretion System such as Found in Vibrio cholerae Depends on the Novel Pilotin AspS
Source: PLoS Pathog. 2013 Jan 10;9(1):e1003117. doi: 10.1371/journal.ppat.1003117 (PMC3542185; doi:10.1371/journal.ppat.1003117)
Supplement: Table S4 — Data collection and refinement statistics. (PDF) [file ppat.1003117.s009.pdf]

Supplementary Table S4: Data collection and refinement statistics.

	V. cholerae AspS
**Data collection**	
Space group	$P2_12_12$
Cell dimensions	
a, *b*, *c* (Å)	34.16, 63.86, 47.54
α, β, γ (°)	90, 90, 90
Resolution (Å)	47.5-1.48 (1.56-1.48)[1]
R_{sym}	0.084 (0.851)
$I / \sigma I$	16.2 (1.73)
Completeness (%)	98.3 (89.0)
Redundancy	7.2 (5.0)
**Refinement**	
Resolution (Å)	47.5-1.48
No. reflections (total/free)	17689/907
R_{work} / R_{free}	0.155/0.176 (0.232/0.248)
No. atoms	
Protein	830
Ligand/ion	15
Water	169
B-factors	
Overall	17.2
Protein	14.7
Ligand/ion	18.5
Water	29.6
Wilson *B*	20.1
R.m.s. deviations	
Bond lengths (Å)	0.013
Bond angles (°)	1.53
Ramachandran distribution (%)[2]	
Favored	99.1
Outliers	0.0

[1]Values in parentheses are for highest-resolution shell.
[2]Calculated using Molprobity.
